# Role of potential bioactive metabolites from traditional Chinese medicine for type 2 diabetes mellitus: An overview

**DOI:** 10.3389/fphar.2022.1023713

**Published:** 2022-11-21

**Authors:** Xiang Li, Jia-Jia Geng-Ji, Yun-Yun Quan, Lu-Ming Qi, Qiang Sun, Qun Huang, Hai-Mei Jiang, Zi-Jian Sun, Hong-Mei Liu, Xin Xie

**Affiliations:** ^1^ State Key Laboratory of Southwestern Chinese Medicine Resources, State Administration of Traditional Chinese Medicine Key Laboratory of Traditional Chinese Medicine Regimen and Health, School of Pharmacy and College of Medical Technology, Chengdu University of Traditional Chinese Medicine, Chengdu, China; ^2^ Translational Chinese Medicine Key Laboratory of Sichuan Province, Sichuan Academy of Chinese Medicine Sciences, Sichuan Institute for Translational Chinese Medicine, Chengdu, Sichuan, China; ^3^ Department of Pharmacy, Personalized Drug Therapy Key Laboratory of Sichuan Province, Sichuan Provincial People’s Hospital, School of Medicine, University of Electronic Science and Technology of China, Chengdu, China; ^4^ Department of Ophthalmology, Hospital of Chengdu University of Traditional Chinese Medicine, Chengdu, China; ^5^ Sichuan Ant Recommendation Biotechnology Co., Ltd., Chengdu, Sichuan, China

**Keywords:** diabetes mellitus, flavonoid, terpenoid, alkaloid, traditional Chinese medicine, bioactive metabolite

## Abstract

Type 2 diabetes mellitus (T2DM) is a metabolic disease with persistent hyperglycemia primarily caused by insulin resistance (IR). The number of diabetic patients globally has been rising over the past decades. Although significant progress has been made in treating diabetes mellitus (DM), existing clinical drugs for diabetes can no longer fully meet patients when they face complex and huge clinical treatment needs. As a traditional and effective medical system, traditional Chinese medicine (TCM) has a unique understanding of diabetes treatment and has developed many classic and practical prescriptions targeting DM. With modern medicine and pharmacy advancements, researchers have discovered that various bioactive metabolites isolated from TCM show therapeutic on DM. Compared with existing clinical drugs, these bioactive metabolites demonstrate promising prospects for treating DM due to their excellent biocompatibility and fewer adverse reactions. Accordingly, these valuable metabolites have attracted the interest of researchers worldwide. Despite the abundance of research works and specialized-topic reviews published over the past years, there is a lack of updated and systematic reviews concerning this fast-growing field. Therefore, in this review, we summarized the bioactive metabolites derived from TCM with the potential treatment of T2DM by searching several authoritative databases such as PubMed, Web of Science, Wiley Online Library, and Springer Link. For the convenience of readers, the content is divided into four parts according to the structural characteristics of these valuable compounds (flavonoids, terpenoids, alkaloids, and others). Meanwhile, the detailed mechanism and future directions of these promising compounds curing DM are also summarized in the related sections. We hope this review inspires increasingly valuable and significant research focusing on potential bioactive metabolites from TCM to treat DM in the future.

## Introduction

Diabetes mellitus (DM) is a chronic disease caused by the relative or absolute lack of insulin or the decreased sensitivity of target cells to insulin, which causes disorders of glucose, lipid, and protein metabolism (Chen et al., 2012; Zheng et al., 2018). The decrease in insulin level and resistance will cause various symptoms, such as hyperglycemia, hyperlipidemia, and hypertension, which will cause a certain degree of damage to organs and the nervous system (Balakumar et al., 2016; Cole and Florez, 2020). These symptoms can seriously exacerbate a patient’s quality of life and even threaten his or her life. According to World Health Organization data, DM was the ninth leading cause of death, with an estimated 1.5 million deaths directly caused by diabetes (World Health Organization, 2021). The International Diabetes Federation statistics indicated that approximately 537 million people aged 20 to 79 would develop diabetes in 2021, meaning that one out of 10 adults was diabetic (International Diabetes Federation, 2021). It is conservatively estimated that the population with diabetes will grow to a horrendous 643 million by 2035 and 783 million by 2045.

Over the past decades, significant progress has been witnessed due to modern pharmacology and clinical medicine advancements, mainly involving insulin injections, oral hypoglycemic drugs, and bariatric surgery in treating DM (Beck et al., 2017; Nguyen and Varela, 2017; Sanchez-Rangel and Inzucchi, 2017). These three methods can reduce blood sugar by improving the body’s glucose metabolism. However, in the face of the growing population of diabetic patients, these classic treatment methods have been challenged immensely and have gradually displayed specific side effects (Nathan, 2015; Ma, 2018). For example, long-term insulin injections will produce insulin resistance (IR) and decrease the body’s endogenous insulin production, enhancing the patient’s dependence on insulin (Yaribeygi et al., 2019). Additionally, although most blood-sugar-lowering drugs can quickly lower blood sugar, some harmful metabolites produced during metabolism can harm the liver, kidneys, and other organs, leading to drug resistance and reduced drug efficacy (Watt et al., 2019; Wu and Ballantyne, 2020). Bariatric surgery shows a better diabetes remission rate despite certain surgical and postoperative risks than drug therapy (Arterburn et al., 2020). However, the strict physical requirements for patients and undesirable clinical sequelae, such as pulmonary embolism, deep vein thrombosis, stomal ulcer, etc., limit its further clinical application (Arterburn et al., 2020). In this context, developing new drugs or methods with few adverse effects, substantial therapeutic effects, and new mechanisms is in high demand.

In the theoretical system of traditional Chinese medicine (TCM), “Xiaoke,” known as one of the common diseases found in the ancient clinical, has symptoms similar to those of DM, including polydipsia, polyphagia, polyuria, and weight loss (Ning et al., 2009). The classic ancient medical books *Yellow Emperor’s Canon of Internal Medicine* and *Synopsis of the Golden Chamber* record that the syndrome (pattern) of Yin deficiency and dryness-heat is the primary factor responsible for the “Xiaoke” disease (Li et al., 2004). TCM theory suggests that the treatment method for Yin deficiency and dryness-heat pathogenesis is to enrich Yin and clear heat (Hongyan et al., 2015). Under the guidance of this clinical theory, many classic and valuable prescriptions for the treatment of DM have been developed successfully, such as Shenqi Wan, and Baihu Jia Renshen Tang (Tong et al., 2012). An effective prescription is usually composed of several medicinal materials under the principles of TCM described in Shen-nong Ben-Cao Jing. Namely, the prescription should feature a strong monarch, accompanied by a minister, assistant, and guide, which leads to increasing and balancing the therapeutic effects of a prescription and producing variants according to the specific severity of the disease and symptom (Xin et al., 2014; Xutian et al., 2014). With the development of modern technology, researchers are gradually unraveling the mysteries of TCM for treating DM. Biological activity experiments have shown several components extracted from TCM, including Rheum palmatum L. (Polygonaceae; Rhei Radix et Rhizoma) and Rheum tanguticum (Maxim. ex Regel) Balf. (Polygonaceae; Rhei Radix et Rhizoma), Coptis chinensis Franch (Ranunculaceae; Coptidis Rhizoma), Bupleurum chinense DC. (Apiaceae; Bupleuri Radix), Rehmannia glutinosa (Gaertn.) DC. (Orobanchaceae; Rehmanniae Radix), Wolfiporia extensa (Peck) Ginns 1984 (Polyporaceae; Poria), etc., show significant effects in the treatment of DM (Tong et al., 2012). Based on significant findings, pharmacologists further explore the magically therapeutic efficacy of these valuable components, such as apigenin, berberine, catalpol, oleanolic acid, crocin, and betaine. These functional components demonstrate few side effects, toxicity, and potential in treating DM. Several reviews focusing on these bioactive molecules’ mechanisms or structural features have been continuously reported over the past decades (Chen et al., 2015; Xu et al., 2018; Li et al., 2019). However, a systematic review of combining the mechanisms and structures of numerous therapeutic molecules isolated from TCM is rarely reported. Therefore, this review summarizes the bioactive metabolites derived from TCM with the potential treatment of DM. These compounds with potential bioactivity to cure DM are divided into four parts (flavonoids, terpenes, alkaloids, and others) according to the structural characteristics of these valuable compounds. Meanwhile, the detailed mechanism of these promising compounds curing DM is also summarized in the related sections. We hope this review will shed some light on related research in treating DM.

## The pathogenesis of diabetes mellitus

Insulin is an essential hormone in glucose metabolism, promoting glucose utilization in the body and thereby reducing blood sugar concentration (Petersen and Shulman, 2018). DM occurs when the body’s blood sugar concentration is too high, and insulin function decreases. According to the degree of insulin dependence, diabetes can be divided into insulin- and non-insulin-dependent diabetes mellitus also called type 1 diabetes mellitus (T1DM) and type 2 diabetes mellitus (T2DM). In addition, there are gestational diabetes and maturity-onset diabetes of the young.

T2DM is the most common type of diabetes, accounting for approximately 90%–95% of diabetes (American Diabetes Asscoiation 2014), and is primarily caused by IR, involving multiple complex mechanisms related to oxidative stress, glucose, and lipid metabolism, inflammation, and immunity. Oxidative stress (OS) refers to an imbalance between free radical production and the antioxidant system, leading to a reduction in insulin sensitivity (i.e., IR) and contributing to the development of T2DM *via* several molecular mechanisms (Yaribeygi et al., 2020). OS is accompanied by excessive production of reactive oxygen species (ROS) and free radicals (Rochette et al., 2014; Rani et al., 2016), which not only have direct deleterious effects but can also indirectly damage cells by activating a variety of stress-sensitive intracellular signaling pathways. OS in T2DM patients plays pivotal roles in the pathophysiology of various complications of diabetes through lipid peroxidation (Farmer and Mueller, 2013; Ito et al., 2019), DNA damage, and mitochondrial dysfunction. Various biomarkers of oxidative stress in T2DM include ROS, malondialdehyde (MDA), total cholesterol and reactive hydroperoxides (ROOH) (Sies and Jones, 2020), catalase (CAT), glutathione peroxidase (GSH-Px), glutathione reductase (GR), and superoxide dismutase (SOD) (Couto et al., 2016; Dehdashtian et al., 2018). Persistent hyperglycemia and obesity are major causes in T2DM patients and play a significant role in developing related metabolic complications, particularly IR (de Heredia et al., 2012; Lisco et al., 2022). Therefore, glucose and lipid metabolism disorder is a pathogenic mechanism in T2DM. Hyperglycemia affects multiple signaling pathways, such as PKC activation, oxidative stress, and TGF-β-SMAD-MAPK signaling, while stimulating advanced glycation end products (AGE) formation caused by altered signaling pathways (Fiorentino et al., 2013). Dyslipidemia is also common in T2DM, such as elevated low-density lipoprotein cholesterol (LDL-C), very low density lipoprotein (VLDL), and triglycerides (TGs), which are associated with IR (Taskinen and Boren, 2015). FOXO1 is a transcription factor in gluconeogenesis and glycogenolysis *via* insulin signaling, which stimulates VLDL overproduction increasing MTTP expression and apoCIII (Guo, 2014). However, insulin cannot inhibit FOXO1 and lipolysis, so dyslipidemia leads to IR. In addition, studies have shown that obesity-induced inflammation can lead to IR and β-cell damage (Lee and Lee, 2014). Adipose tissue is responsible for cytokine production and other bioactive substances during inflammation, including tumor necrosis factor-α (TNF-α), IL-1, IL-6, and IL-10, leptin, resistin, etc. (Zatterale et al., 2020). Not only is adipose tissue a major source of inflammatory markers, but almost all mechanisms involved in T2DM or associated complications are related to the inflammatory response. In pancreatic islets, elevated blood glucose, increased ROS formation etc. all promote the activation of the inflammasome, thus enabling the production of mature interleukin-1β (IL-1β) (Lontchi-Yimagou et al., 2013; Donath, 2014). The pathogenesis of T2DM is thought to be related to innate and adaptive immunity, as the proliferation of T cells and macrophages is altered, and the function of NK cells and B cells is impaired in patients with T2DM (Zhou et al., 2018). In addition, a decrease in insulin receptors is also a cause of IR because insulin works by binding to insulin receptors on cell membranes (Guo, 2014). After insulin receptor activation and autophosphorylation, it triggers the phosphorylation of insulin receptor substrate proteins, such as IRS-1 and IRS-2. The IRS protein plays a vital role in the insulin signaling pathway. For example, it can activate the PI3K-AKT pathway to regulate glucose uptake and suppress gluconeogenesis (Zeyda and Stulnig, 2009). Therefore, insulin substrate proteins are an important target for treating diabetes. In addition, DM also causes a series of complications that affect the quality of life of diabetic patients, such as stroke, diabetic retinopathy, diabetic neuropathy, heart attack, diabetic nephropathy, diabetic angiopathy, and diabetic plexopathy (Bhupathiraju and Hu, 2016; Napoli et al., 2017; Targher et al., 2018; Zheng et al., 2018). In general, oxidative stress, inflammation, immunity, hyperglycemia, dyslipidemia, and IR play an essential role in the pathogenesis of diabetes.

## The classical formulas for diabetes mellitus

**FIGURE 1:**
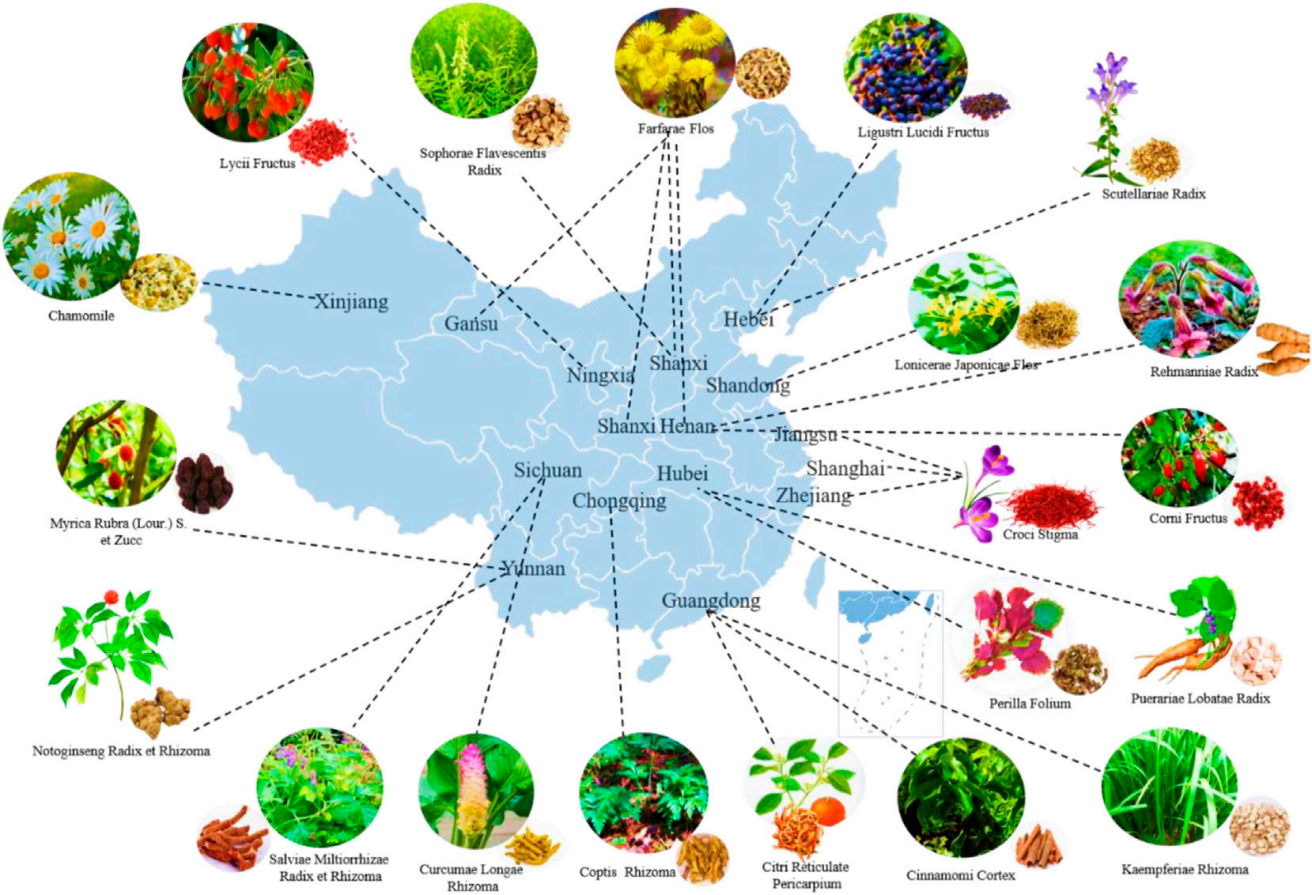
The distribution of Chinese medicine for T2DM in the main producing area of China.

**FIGURE 2:**
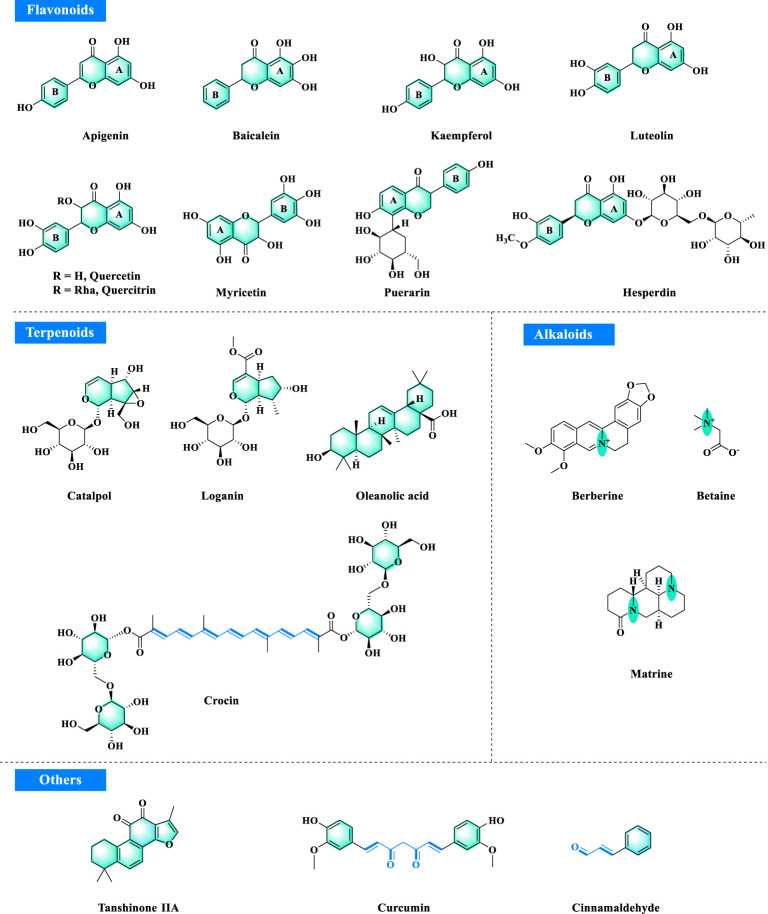
Potential bioactive metabolites from TCM in T2DM.

Classical formulas are a class of TCM prescriptions that have been historically tested and can effectively treat diseases. The famous physician Zhang Zhongjing set up a particular chapter on “diabetes disease” in *Synopsis of the Golden Chamber* and listed two formulas for DM, namely Baihu Jia Renshen Tang for excess heat in the lung and stomach and Shenqi Wan for the kidney Qi deficiency syndrome (Zhang, 2014). Inspired by Zhang Zhongjing’s formulas, many derived classical prescriptions were born accordingly (Table 1). The derived prescriptions can be divided into several types of classical prescriptions that utilize the primary medicines of Huanglian [Coptis chinensis Franch. (Ranunculaceae; Coptidis Rhizoma)], Dahuang [Rheum palmatum L. (Polygonaceae; Rhei Radix et Rhizoma); Rheum tanguticum (Maxim. ex Regel) Balf. (Polygonaceae; Rhei Radix et Rhizoma)], Chaihu (Bupleurum chinense DC. [Apiaceae; Bupleuri Radix]), Fuling [Wolfiporia extensa (Peck) Ginns 1984 (Polyporaceae; Poria)], Huangqi [*Astragalus mongholicus* Bunge (Fabaceae; Astragali Radix)], etc., respectively (Pang and Ni, 2019). The formula for Huanglian is a prescription with Coptis chinensis Franch. (Ranunculaceae; Coptidis Rhizoma) as the primary medicine, which can clear dampness and heat in the stomach and intestines (Pang et al., 2015). It is mainly used to treat diabetic patients with a fatty diet and excessive drinking (Pang and Ni, 2019). Representative prescriptions include Gegen Qinlian Tang, Banxia Xiexin Tang, Dahuang Huanglian Xiexin Tang, Ganjiang Huangqin Huanglian Ginseng Tang, Wumei Wan, etc. (Tong et al., 2011). The formula for Dahuang refers to the prescription of Rheum palmatum L. (Polygonaceae; Rhei Radix et Rhizoma) and Rheum tanguticum (Maxim. ex Regel) Balf. (Polygonaceae; Rhei Radix et Rhizoma) as the primary medicine, which can clear the internal organs, purify the turbidity, and treat the “Zhong Man” of T2DM, with the main symptoms of constipation, abdominal distension, and bad breath (He and Ni, 2021). Representative prescriptions include Houpo Sanwu Tang, Da/Xiao Cheng Qi Tang, Yin Chen Hao Tang, etc. The formula for Chaihu is a prescription with Bupleurum chinense DC. (Apiaceae; Bupleuri Radix) as the primary medicine, which can relieve depression and dissipate stagnation, and treat DM with the main symptoms of chest fullness, irritability, bitter mouth, and insomnia (He and Ni, 2021). Clinically, Chaihu can relieve emotional depression, and related prescriptions include Xiao Chaihu Tang and Sini San (Wang, 2011). The formula about Huangqi refers to the prescriptions with *Astragalus mongholicus* Bunge (Fabaceae; Astragali Radix) as the primary medicine, which can invigorate Qi and deficiency and treat DM and its complications with the main symptoms of fatigue, shortness of breath, inconvenience in urination, and dull complexion (Pang and Ni, 2019). Huangqi Jianzhong Tang, Huangqi Guizhi Wuwu Tang, Fangji Huangqi Tang, and Yupingfeng San are the representative prescriptions (Yin et al., 2021). Further experiments showed that the active ingredients (flavonoids, terpenes, alkaloids, etc.) extracted from the single botanical drug in the classic formulas have a good antidiabetic effect, which indirectly proves that TCM has good potential for the treatment of diabetes. The distribution of TCM used to treat T2DM in China’s principal producing areas is shown in Figure 1, and the potential bioactive metabolites from TCM are shown in Figure 2 and Table 2.

**TABLE 1:** The composition, dosage, and extraction method of classical formulas.

Classical formulas	Composition and dosage	Extraction method	References
Baihu Jia Renshen Tang	Zhimu 18 g, *Anemarrhena asphodeloides* Bunge rhizomes	Water decoction	Cheng and Chen, (2017)
	Shigao 50 g, Gypsum Fibrosum (mineral drug)		
	Gancao 6 g, *Glycyrrhiza uralensis* Fisch. ex DC. roots and rhizomes		
	Jingmi 9 g, Japonica rice (seeds)		
	Renshen 9 g, *Panax ginseng* C. A. Mey roots		
Shenqi Wan	Gan Dihuang 24 g, *Rehmannia glutinosa* (Gaertn.) DC. root tubers	Water decoction	
	Shuyu 12 g, *Dioscorea oppositifolia* L. rhizomes		
	Shanzhuyu 12 g, *Cornus officinalis* Siebold and Zucc. fruits		
	Zexie 9 g, *Alisma plantago-aquatica subsp. orientale* (Sam.) Sam. tuber		
	Fuling 9 g, *Poria cocos* (Schw.) Wolf		
	Mudanpi 9 g, *Paeonia × suffruticosa* Andrews barks		
	Guizhi 3 g, *Neolitsea cassia* (L.) Kosterm. shoots		
	Prepared Fuzi 3 g, *Aconitum carmichaeli* Debeaux roots		
Gegen Qinlian Tang	Gegen 15 g, *Pueraria montana* var. lobate (Willd.) roots	Water decoction	
	Zhi Gancao 6 g, *Glycyrrhiza uralensis* Fisch. ex DC. roots and rhizomes		
	Huangqin 9 g, *Scutellaria baicalensis* Georgi roots		
	Huanglian 9 g, *Coptis chinensis* Franch. rhizomes		
Banxia Xiexin Tang	Banxia 12 g, *Pinellia ternata* (Thunb.) Makino tubers	Water decoction	
	Huangqin 9 g, *Scutellaria baicalensis* Georgi roots		
	Ganjiang 9 g, *Zingiber officinale* Roscoe rhizomes		
	Renshen 9 g, *Panax ginseng* C. A. Mey roots		
	Huanglian 3 g, *Coptis chinensis* Franch. rhizomes		
	Zhi Gancao 9 g, *Glycyrrhiza uralensis* Fisch. ex DC. roots and rhizomes		
	Dazao 6 g, *Ziziphus jujuba* Mill. fruits		
Wumei Wan	Wumei 300 pieces, *Prunus mume* (Siebold) Siebold and Zucc. fruits	Water decoction	
	Xixin 18 g, *Asarum heterotropoides* F.Schmidt roots and rhizomes; *Asarum sieboldii* Miq. roots and rhizomes		
	Ganjiang 30 g, *Zingiber officinale* Roscoe rhizomes		
	Huanglian 48 g, *Coptis chinensis* Franch. rhizomes		
	Danggui 12 g, *Angelica sinensis* (Oliv.) Diels roots		
	Prepared Fuzi 18 g, *Aconitum carmichaeli* Debeaux roots		
	Huajiao 5 g, *Zanthoxylum bungeanum* Maxim. peels		
	Guizhi 18 g, *Neolitsea cassia* (L.) Kosterm. shoots		
	Renshen 18 g, *Panax ginseng* C. A. Mey roots		
	Huangbai 18 g, *Phellodendron chinense* C.K.Schneid. barks		
	Shujiao 12 g, *Zanthoxylum bungeanum* Maxim. peels		
Houpo Sanwu Tang	Houpo 24 g, *Magnolia officinalis* Rehder and E. H. Wilson barks	Water decoction	
	Zhishi 9 g, *Citrus × aurantium* L. fruits		
	Dahuang 12 g, *Rheum palmatum* L. roots and rhizomes; *Rheum tanguticum* (Maxim. ex Regel) Balf. roots and rhizomes		
Da Chengqi Tang	Dahuang 12 g, *Rheum palmatum* L. roots and rhizomes; *Rheum tanguticum* (Maxim. ex Regel) Balf. roots and rhizomes	Water decoction	
	Houpo 20 g, *Magnolia officinalis* Rehder and E. H. Wilson barks		
	Zhishi 15 g, *Citrus × aurantium* L. fruits		
	Mangxiao 6 g, Natrii Sulfas (mineral drugs)		
Xiao Chengqi Tang	Dahuang 12 g, *Rheum palmatum* L. roots and rhizomes; *Rheum tanguticum* (Maxim. ex Regel) Balf. roots and rhizomes	Water decoction	
	Houpo 6 g, *Magnolia officinalis* Rehder and E. H. Wilson barks		
	Zhishi 15 g, *Citrus × aurantium* L. fruits		
Yin Chen Hao Tang	Yinchen 18 g, *Artemisia capillaris* Thunb. herb; *Artemisia scoparia* Waldst. and Kit. herb	Water decoction	
	Zhizi 12 g, *Gardenia jasminoides* J. Ellis fruits		
	Dahuang 6 g, *Rheum palmatum* L. roots and rhizomes; *Rheum tanguticum* (Maxim. ex Regel) Balf. roots and rhizomes		
Xiao Chaihu Tang	Chaihu 24 g, *Bupleurum chinense* DC. roots	Water decoction	
	Huangqin 9 g, *Scutellaria baicalensis* Georgi roots		
	Renshen 9 g, *Panax ginseng* C. A.Mey roots		
	Gancao 9 g, *Glycyrrhiza uralensis* Fisch. ex DC. roots and rhizomes		
	Banxia 9 g, *Pinellia ternata* (Thunb.) Makino tuber		
	Shengjiang 9 g, *Zingiber officinale* Roscoe rhizomes		
	Dazao 6 g, *Ziziphus jujuba* Mill. fruits		
Sini San	Zhi Gancao 9 g, *Glycyrrhiza uralensis* Fisch. ex DC. roots and rhizomes	Water decoction	
	Zhishi 9 g, *Citrus × aurantium* L. fruits		
	Chaihu 9 g, *Bupleurum chinense* DC. roots		
	Baishao 9 g, *Paeonia lactiflora* Pall. roots		
Huangqi Jianzhong Tang	Guizhi 9 g, *Neolitsea cassia* (L.) Kosterm. shoots	Water decoction	
	Zhi Gancao 6 g, *Glycyrrhiza uralensis* Fisch. ex DC. roots and rhizomes		
	Dazao 6 g, *Ziziphus jujuba* Mill. fruits		
	Shaoyao 18 g, *Paeonia lactiflora* Pall. roots		
	Shengjiang 9 g, Zingiber officinale Roscoe rhizomes		
	Jiaoyi 30 g, maltose		
	Huangqi 5 g, *Astragalus mongholicus* Bunge roots		
Huangqi Guizhi Wuwu Tang	Huangqi 9 g, *Astragalus mongholicus* Bunge roots	Water decoction	
	Shaoyao 9 g, *Paeonia lactiflora* Pall. roots		
	Guizhi 9 g, *Neolitsea cassia* (L.) Kosterm. shoots		
	Shengjiang 18 g, Zingiber officinale Roscoe rhizomes		
	Dazao 6 g, *Ziziphus jujuba* Mill. fruits		
Fangji Huangqi Tang	Fangji 12 g, *Stephania tetrandra* S. Moore roots	Water decoction	
	Gancao 6 g, *Glycyrrhiza uralensis* Fisch. ex DC. roots and rhizomes		
	Baizhu 9 g, *Atractylodes macrocephala* Koidz. rhizomes		
	Huangqi 15 g, *Astragalus mongholicus* Bunge roots		
Yupingfeng San	Fangfeng 15 g, *Saposhnikovia divaricata* (Turcz. ex Ledeb.) Schischk. roots	Water decoction	
	Zhi Huangqi 30 g, *Astragalus mongholicus* Bunge roots		
	Baizhu 30 g, *Atractylodes macrocephala* Koidz. rhizomes		

**TABLE 2:** The family and species of bioactive metabolites from TCM (Kew Science, 2022).

Bioactive metabolites	Family	*Species*	Medicinal parts
Puerarin	Fabaceae	*Pueraria montana var. lobata* (Willd.) Maesen and S.M.Almeida ex Sanjappa and Predeep	Puerariae Lobatae Radix
Quercetin	Araliaceae	*Panax notoginseng* (Burk.) F. H. Chen	Notoginseng Radix et Rhizoma
	Asteraceae	*Tussilago farfara* L	Farfarae Flos
	Cupressaceae	*Platycladus orientalis* (L.) Franco	Platycladi Cacumen
Luteolin	Caprifoliaceae	*Lonicera japonica* Thunb	Lonicerae Japonicae Flos
	Lamiaceae	*Perilla frutescens* (L.) Britton	Perillae Folium
Kaempferol	Zingiberaceae	*Kaempferia galanga* L	Kaempferiae Rhizoma
Hesperidin	Rutaceae	*Citrus × aurantium* L	Citri Reticulatae Pericarpium
Baicalein	Lamiaceae	*Scutellaria baicalensis* Georgi	Scutellariae Radix
Catalpol	Orobanchaceae	*Rehmannia glutinosa* (Gaertn.) DC.	Rehmanniae Radix
Oleanolic acid	Oleaceae	*Ligustrum lucidum* W.T.Aiton	Ligustri Lucidi Fructus
Crocin	Iridaceae	*Crocus sativus* L	Croci Stigma
Loganin	Cornaceae	*Cornus officinalis* Siebold and Zucc	Corni Fructus
Berberine	Ranunculaceae	*Coptis chinensis* Franch	Coptidis Rhizoma
Matrine	Fabaceae	*Sophora flavescens* Aiton	Sophorae Flavescentis Radix
Betaine	Solanaceae	*Lycium barbarum* L	Lycii Fructus
Curcumin	Zingiberaceae	*Curcuma longa* L	Curcumae Longae Rhizoma
Cinnamaldehyde	Lauraceae	*Neolitsea cassia* (L.) Kosterm	Cinnamomi Cortex
Tan IIA	Lamiaceae	*Salvia miltiorrhiza* Bunge	Salviae miltiorrhizae radix et rhizoma

## Flavonoids

Flavonoids generally refer to compounds in which two benzene rings (A ring and B ring) with phenolic hydroxyl groups are connected through the central three carbon atoms (C6-C3-C6 units). The structure is often substituted with phenolic hydroxyl, methoxy, methyl, isopentenyl, and other functional groups. The flavonoids summarized in this review include apigenin, baicalein, puerarin, quercetin, hesperidin, myricetin, kaempferol (Table 3) and have diverse physiological activities, such as antioxidant, anti-inflammatory, hypoglycemic, hepatoprotective, antibacterial, antiviral, and antitumor effects. Flavonoids can enhance the activity of antioxidant enzymes by inhibiting the generation of ROS, free radicals, and MDA. For example, apigenin can enhance the activity of antioxidant enzymes by neutralizing ROS (Wang et al., 2014). Flavonoids also demonstrate anti-inflammatory effects by inhibiting the activities of nuclear factor kappa-B (NF-κB) and TNF-α or by activating Adenosine 5‘-monophosphate (AMP)-activated protein kinase (AMPK). For instance, kaempferol can exert anti-inflammatory effects by inhibiting the activities of NF-κB and TNF-α (Calderon-Montano et al., 2011). Moreover, flavonoids can reduce blood sugar by regulating AMPK activity, regulating Peroxisome proliferator-activated receptor *γ* (PPARγ), and inhibiting α-Glucosidase (α-Glu) activity. For example, hesperidin can inhibit α-Glu activity and starch digestion and reduce blood glucose concentrations (Shen et al., 2012).

**TABLE 3:** Representative antidiabetic flavonoids.

Bioactive metabolites	Detail	Cell lines/model	Dose	Negative/positive control	Duration	References
Apigenin	Neutralized active oxygen and enhanced the activity of antioxidant enzymes to achieve anti-oxidation	Alloxan-induced diabetic mice	0.78 mg/kg	NC: saline (0.1 ml)	10 days	Panda and Kar, (2007)
Baicalein	Activated AMPK to achieve anti-inflammatory function and inhibit IR	HFD-induced mice	400 mg/kg	—	29 weeks	Pu et al. (2012)
	Downregulated related genes SREBP-1c, FAS, key enzymes for cholesterol and fatty acid synthesis to regulate lipid metabolism	HFD-induced Rats	80 mg/kg	NC: saline	18 weeks	Guo et al. (2009)
Puerarin	Inhibited NOX4, NOX2, and NF-κB	STZ-induced diabetic rats	18, 45 mg/kg	—	3 weeks	Li et al. (2016)
	Reduced ROS and renewed the activities of antioxidant enzymes	Lead-induced Rats	200 mg/kg	NC: saline (0.9% NaCl)	75 days	Liu et al. (2011)
	Upregulated the mRNA levels of endogenous antioxidant enzymes	Preadipocyte 3T3-L1 cell	100 μM	—	6 days	Lee K. M et al. (2010)
	Inhibitied NF-κB-driven liver inflammation and the TGF-β/smad signaling pathway	HFD-induced diabetic rats	100 mg/kg	—	8 weeks	Hou et al. (2018)
	lower blood glucose levels	STZ-induced diabetic rats	15 mg/kg	NC: saline	2 weeks	Hsu et al. (2003)
	Decreased the blood glucose level of T2DM by downregulating ADRP mRNA expression and depressing IR	HFD-induced Diabetic Rats	40, 80, and 160 mg/kg	NC: saline	6 weeks	Sun et al. (2008)
	Activated GLP-1R signaling	HFD-induced Diabetic Mice	150 mg/kg	—	35 days	Yang et al. (2016)
	Reduced pancreatic tissue damage and upregulated IRS-1 and IGF-1	STZ-induced diabetic mice	20, 40, and 80 mg/kg	PC: metformin (40 mg/kg)	14 days	Wu et al. (2013)
Quercetin	Inhibited the generation of free radicals, enhanced the activity of antioxidant enzymes, and reduced the content of MDA and NO	STZ-induced broilers	0.2, 0.4, and 0.6 g/kg	—	42 days	Ying et al. (2020)
	Promoted insulin secretion through the ERK1/2 pathway	INS-1 cells	20 μM	—	5 days	Youl et al. (2010)
	Inhibited COX and LOX	RLE cells	10, 20 μM	—	24 h	Lee O. H et al. (2010)
	Blocked the production of pro-inflammatory cytokines	Orbital fat tissues	100 μM	—	72 h	Yoon et al. (2012)
	Activated AMPK pathway to inhibit adipogenesis	3T3-L1 cells	0, 10, 50, and 100 μM	—	8 days	Ahn et al. (2008)
Quercitrin	Restored glycogen content and carbohydrate metabolic enzyme activities	STZ-induced diabetic rats	30 mg/kg	NC: carboxymethyl cellulose (1 ml/rat)	30 days	Babujanarthanam et al. (2010)
	Decreased fasting blood glucose, increased insulin levels, and improved the antioxidant status	STZ-induced diabetic rats	30 mg/kg	NC: carboxymethyl cellulose (1 ml/rat)	30 days	Babujanarthanam et al. (2011)
Hesperidin	Inhibited the generation of ROS and reduced the content of MDA	Exhausted exercise model in rats	200 mg/kg	—	52 days	Estruel-Amades et al. (2019)
	Inhibited cell apoptosis by reducing the levels of Caspase-9, Caspase-3, and Bax/Bcl-2	The retinal ganglion cell 5 (RGC-5) cells	12.5, 25, and 50 μM	—	48 h	Liu et al. (2017)
	Suppression of systemic levels of IL-6, MCP-1, and TBARS	HFD-induced C57BL/6J mice	100 mg/kg	—	4 weeks	Ferreira et al. (2016)
	Inhibited both amylose and amylopectin digestion	HepG2 cells	10, 50 μM	PC: Metformin (1,000 μM)	24 h	Shen et al. (2012)
	Inhibited hepatic gluconeogenesis	Rat liver	40, 80, 120, 160, and 200 μM	—	24 h	Zareei et al. (2017)
	Increased PPAR-γ activity or PPAR-γ mRNA expression	HEK293 cells	400 and 800 μg/ml EMS	—	9 weeks	Shin et al. (2013)
	Improved the impaired fasting glucose level and glucose tolerance	HFD-induced mice	1%, 5% EMS (ethanol extract of Prunus mume fruits)	—	9 weeks	Shin et al. (2013)
Myricetin	Increased GSH level and CAT activity	Adult female Wistar Albino mice	3 mg/kg	—	7 days	Hassana et al. (2017)
	Reduced MDA and oxidation protein	MC3T3-E1 cell	0.01–10 μM	—	2 days	Lee and Choi, (2008)
	Inhibition of NF-κB-mediated inflammatory responses	LPS-induced mice	120 mg/kg	PC: dexamethasone (0.5 mg/kg)	4 h	Qi et al. (2017)
	Inhibited α-Amylase (α-Amy) and α-Glucosidase (α-Glu) activity	Pancreatic α-Amylase, α-Glucosidase	0.5, 1 μg/ml	—	20 min	Meng et al. (2016)
Kaempferol	Inhibited the production of ROS, enhanced the activity of antioxidant enzymes, and scavenged free radicals	dRib-induced HIT-T15 cells	10 μM	—	30 min	Lee Y. Jet al. (2010)
	Inhibited the activation of AP-1	PC3 cells	50 μM	—	24 h	Gopalakrishnan et al. (2006)
	Ameliorated hyperglycemia as ligands of PPARγ	3T3-L1 adipocytes	5, 10, 20, and 50 μM	PC: Rosiglitazone (1 μM)	7 days	Fang et al. (2008)
	Reduced inflammation by downregulation of IKK and inhibition of NF-κB pathway activation	HFD-Induced Rats	50, 150 mg/kg	PC: Aspirin (100 mg/kg)	9 weeks	Luo et al. (2015)
	Protected pancreatic beta-cell survival and function	INS-1E beta-cells	10 μM	—	4 days	Zhang and Liu, (2011)
Luteolin	Reduced oxidative stress and enhanced activity in the NOS–NO pathway	STZ-induced diabetic rats	10, 50, 100 mg/kg	—	8 weeks	Qian et al. (2010)
	Attenuated IR through the activation of AMPKα1 signaling	HFD-induced diabetic mice	0.01% v/v	—	20 weeks	Zhang et al. (2016)
		RAW 264.7 cells	20 μM	—	24 h	
	Reduced oxidative stress by elevating the enos expression, enhancing mnsod, and suppressing mptp in the downstream	STZ-induced diabetic rats	100 mg/kg	—	2 weeks	Zhou et al. (2021)
	Reduced oxidative stress by elevating the eNOS expression, enhancing MnSOD, and suppressing mPTP downstream	STZ-induced diabetic rats	50 mg/kg	—	2 weeks	Yang et al. (2015)
	Increased the antioxidant capacity such as GSH and gpx activity, and decreased MDA level	STZ-induced diabetic rats	25, 50, 100 mg/kg	NC: 0.5% CMC	12 weeks	Chen Y et al. (2017)
	Inhibited IL-1β, vascular endothelial growth factor, and NF-κB mRNA and protein expression					
	Decreased the IL-1β mRNA expression and TNF-α mRNA expression to ameliorate inflammatory	STZ-induced diabetic rats	50, 100 mg/kg	—	8 weeks	Gu et al. (2018)

### Puerarin

Puerarin is a flavonoid that mainly exists in the TCM *Pueraria montana* var. Lobata (Willd.) Maesen and S.M.Almeida ex Sanjappa and Predeep (Fabaceae; Puerariae Lobatae Radix), which has been used to treat DM in China since the 1990s (Zhou et al., 2014). According to research, puerarin plays an essential role in antioxidation. For example, it has been reported that oral puerarin (18, 45 mg/kg/day) can significantly reduce serum H_2_O_2_ and NO levels, inhibit the expression of NOX2 and NOX4, and regulate NF-κB in STZ-induced diabetic rats (Li et al., 2016). Moreover, most studies point out that puerarin can achieve its antioxidant function by changing the level of enzymes related to the oxidation process. Specifically, first, puerarin can protect islets from oxidative stress induced by hydrogen peroxide by increasing peroxidase and SOD (Xie and Du, 2011). Second, studies have shown that treatment with puerarin (100 μM) can upregulate the mRNA levels of endogenous antioxidant enzymes in 3T3-L1 preadipocytes so that they increase glucose-6-phosphate dehydrogenase (G6PDH), GR and hydrogen peroxide enzyme expression (Lee O.-H. et al., 2010). Third, puerarin (200 mg/kg) can improve lead-induced liver injury and hyperlipidemia by downregulating ROS production, upregulating antioxidant enzyme activity, and reducing liver lipid synthesis by upregulating metabolic gene expression in lead-induced rats (Liu et al., 2011). Some reports have demonstrated that puerarin possesses anti-inflammatory activity. Puerarin (100 mg/kg/day) can reduce liver damage by inhibiting NF-κB-driven liver inflammation and the TGF-β/smad signaling pathway in HFD-induced diabetic rats (Hou et al., 2018). Elevated blood sugar is an essential factor in diabetes, and puerarin can effectively regulate blood sugar; for example, intravenous puerarin (15 mg/kg) can lower blood glucose levels in STZ-induced diabetic rats (Hsu et al., 2003). Puerarin also significantly enhances glucose uptake by insulin-sensitive cells and reduces blood glucose levels (Zhou et al., 2014). At the same time, it is reported that puerarin can regulate lipid metabolism. In the adipose tissue of T2DM rats, puerarin (40, 80, and 160 mg/kg) significantly blocked the mRNA expression of the adipose differentiation-related protein (ADRP) gene (Sun et al., 2008).

Furthermore, studies have pointed out that puerarin can promote glucose uptake of adipocytes by promoting PPARγ expression and enhancing the differentiation of preadipocytes (Xu et al., 2005). Puerarin affects pancreatic islet cells to influence the development of DM. In high-fat-diet (HFD) induced and db/db diabetic mice, puerarin (150 mg/kg) increases pancreatic β-cell mass and proliferation by promoting β-cell survival and inhibiting apoptosis, and increasing insulin secretion *via* activation of GLP-1R signaling (Yang et al., 2016). Different dosages of puerarin (20, 40, and 80 mg/kg) administration effectively reduce pancreatic tissue damage and upregulate intrapancreatic protein levels of insulin receptor substrate-1 (IRS-1) and insulin-like growth factor-1 (IGF-1) in STZ-induced diabetic mice (Wu et al., 2013). In addition, puerarin can increase the activity of antioxidative stress-related enzymes (such as CAT and SOD) and scavenge ROS to protect pancreatic islets and β-cells (Chen X. et al., 2018).

### Quercetin

Quercetin is a flavonoid widely distributed in TCM, including Panax notoginseng (Burkill) F.H.Chen (Araliaceae; Notoginseng Radix et Rhizoma), Tussilago farfara L. (Asteraceae; Farfarae Flos), Platycladus orientalis (L.) Franco (Cupressaceae; Platycladi Cacumen), etc., has been shown to play a curative effect in DM. Quercetin has two antioxidant groups in the quercetin molecule, namely the catechol group in the B ring and the OH group at the third position of the A ring, which exerts its antioxidant activity by inhibiting free radicals and increasing oxidase activity (D'Andrea, 2015). Quercetin can prevent the formation of free radicals by preventing the spread of lipid peroxidation and increasing glutathione levels. In the STZ-induced broiler model, quercetin (0.2, 0.4, and 0.6 g/kg) can regulate glucose metabolism and reduce oxidative damage by increasing the activity of antioxidant enzymes, reducing the levels of MDA and NO, and activating the expression of genes related to PI3K/PKB (Ying et al., 2020). Studies have shown that quercetin (20 μmol/L) can protect β-cells from oxidative damage and promote insulin secretion through the ERK1/2 pathway in INS-1 cells (Youl et al., 2010). In addition, quercetin can protect cells suffering from oxidative stress by preventing Ca^2+^-dependent cell death. Quercetin has proven to be a powerful anti-inflammatory weapon. Although the anti-inflammatory mechanism of quercetin is still unclear, researchers have carried out many explorations. Its anti-inflammatory mechanism has increased antioxidant activity, modulated NF-κB, and reduced pro-inflammatory enzyme activity and cytokine levels (Chen et al., 2016). Studies have demonstrated that quercetin (10, 20 μM) can inhibit the production of COX-2 and PGE_2_ by targeting PI3K in RLE cells (Lee K. M. et al., 2010). In primary orbital fibroblasts and tissue culture, quercetin (100 μM) can block the production of proinflammatory cytokines (Yoon et al., 2012). In addition, quercetin plays a vital role in glucose and lipid metabolism. Quercetin (0, 10, 50, and 100 μM) can block adipogenesis by stimulating the mitogen-activated protein kinase (MAPK) signaling pathway and induce mature adipocyte apoptosis by controlling the ERK and JNK pathways in 3T3-L1 preadipocytes (Ahn et al., 2008; Chen et al., 2016). Quercetin is a flavonoid glycoside, which becomes quercetin after hydrolysis. Quercitrin also has an antidiabetic effect similar to that of quercitrin. In STZ-induced diabetic rats, quercitrin (30 mg/kg) orally administered for 30 days significantly decreased fasting blood glucose, increased insulin levels, and improved the antioxidant status of diabetic rats by reducing lipid peroxidation products and increasing antioxidants (Babujanarthanam et al., 2011). These results suggest that quercetin has antioxidant effects in STZ-induced experimental DM. In another experiment of STZ-induced diabetic rats, oral administration of quercitrin (30 mg/kg) for 30 days caused decreased blood glucose, increased insulin levels, and restored glycogen content and carbohydrate metabolic enzyme activities (Babujanarthanam et al., 2010). This result suggests that quercetin positively impacted glucose metabolism in diabetic rats.

### Luteolin

Luteolin, a natural flavonoid, is beneficial in treating DM, found in TCM such as *Lonicera japonica* Thunb. (Caprifoliaceae; Lonicerae Japonicae Flos), Perilla frutescens (L.) Britton (Lamiaceae; Perillae Folium), etc. It is demonstrated that luteolin has an antioxidant effect through the NOS/NO pathway and Nrf2-related antioxidative signaling pathway. The NOS/NO pathway plays a vital role in oxidative stress. Luteolin (10, 50, and 100 mg/kg) decreased ROS level and OH− • formation and increased level of NO and NOS, and SOD activity in rat aorta of STZ-induced diabetic rats, which proved that luteolin reduced oxidative stress by upregulating activity in NOS–NO pathway (Qian et al., 2010). In STZ-induced diabetic rats, luteolin (50 mg/kg) reduced oxidative stress by elevating the eNOS expression, enhancing MnSOD, and suppressing mPTP downstream to protect the diabetic heart after ischemia/reperfusion (Yang et al., 2015). Moreover, the nuclear factor erythroid 2-related factor 2 (Nrf2) is critical in ameliorating oxidative stress. Luteolin (100 mg/kg) elevated the levels of antioxidant enzymes (e.g., SOD, GSH), reduced MDA, and enhanced nuclear Nrf2 and the Nrf2-related antioxidative signaling pathway attenuated cardiac ischemia/reperfusion injury in STZ-induced diabetic rats (Chen Y. et al., 2017). Luteolin (100 mg/kg) ameliorated the expression of antioxidant genes regulated by Nrf2, reduced MDA, and blocked sestrin2 transcription in the diabetic I/R hearts, suggesting that Nrf2 and sestrin2 stimulated antioxidative effects in STZ-induced rats (Xiao et al., 2019; Zhou et al., 2021). The anti-inflammatory effect of luteolin is also crucial to the treatment of DM by inhibiting the expression of inflammatory cytokines, NF-κB and TNF-α. In STZ-induced diabetic rats, luteolin (25, 50, and 100 mg/kg) suppressed IL-1β, vascular endothelial growth factor, NF-κB mRNA, and protein expression (Gu et al., 2018). Treatment with luteolin (50, 100 mg/kg) decreased the IL-1β mRNA and TNF-α mRNA expression in the hippocampus, thereby ameliorating inflammation, suggesting an anti-inflammatory effect of luteolin (Gu et al., 2018). Glucose and lipid metabolism are closely related to DM, and luteolin can modulate glucose and lipid metabolism by upregulating LXRα expression and downregulating of FAS and SREBP-1c expression. LXRα is a nuclear receptor that affects lipid and cholesterol metabolism (Christopherson et al., 2009). Luteolin (0.005%, w/w) suppressed hepatic lipogenesis and lipid absorption and simultaneously increased adipocyte PPARγ protein expression to control lipid metabolism in HFD-induced mice (Kwon et al., 2015). In addition, luteolin ameliorated IR through TNF-α, SREBP1 expression, and PI3K signaling. TNF-α is one of the proinflammatory cytokines that can directly affect beta-cell function. Both *in vivo* and *in vitro* studies indicated that luteolin attenuated IR through the activation of AMPKα1 signaling in HFD-induced mice and RAW 264.7 cells (Zhang et al., 2016). Decreased expression of Irs2 can lead to IR, while Irs2 is negatively feedback-regulated by SREBP1, and luteolin improves hepatic insulin sensitivity by inhibiting SREBP1 expression in HFD-induced mice (Kwon et al., 2015).

### Myricetin

Myricetin, a natural flavonoid, is abundantly found in plants which can be extracted in Myrica rubra (Lour.) Siebold and Zucc. (Myricaceae)*.* Myricetin possesses antidiabetic properties, such as anti-antioxidant, anti-inflammatory, regulation of glucose and lipid metabolism, and protection of islets. Myricetin can reduce the oxidative damage of diabetes-related bone diseases. In 2-deoxy-D-ribose-induced osteoblasts MC3T3-E1 cells, myricetin (10^−9^–10^–5^ M) increased the survival rate of cells, reduce the production of MDA protein carbonyl, and advanced the oxidation of protein (Lee and Choi, 2008). Myricetin also blocks oxidative stress by increasing the activity of antioxidant enzymes (SOD, CAT, etc.) (Semwal et al., 2016). For example, myricetin (3 mg/kg) significantly increased GSH levels and CAT activity in kidney tissues (Hassana et al., 2017). In isolated rat liver nuclei, myricetin was found to have pro-oxidant properties, and it can induce nuclear DNA degradation that coincides with lipid peroxidation, which can be enhanced by the action of copper (II) and iron (III) (Sahu and Gray, 1993; Semwal et al., 2016). Myricetin was found to have good anti-inflammatory activity. Studies have pointed out that oxidative stress activates multiple inflammatory mediators associated with several chronic diseases, and it is known from the preceding that myricetin can block oxidative stress by increasing the activity of antioxidant enzymes (Song et al., 2021). In addition, studies revealed that myricetin possessed potential effects on inflammatory osteolysis through the regulation of RANKL-related signals (Wang et al., 2018). Glucagon-like peptide-1 (GLP-1) is an effective potential target for treating type 2 diabetes, stimulating insulin secretion, and regulating blood sugar levels (Li et al., 2017). Myricetin can act as a GLP-1 receptor (GLP-1R) agonist, and studies verified that long-term oral administration of myricetin could regulate glucose metabolism (Li et al., 2017). α-Amylase (α-Amy) and α-Glu are two enzymes involved in starch hydrolysis, which can promote an increase in blood sugar. Studies have shown that myricetin (0.5, 1 μg/ml) inhibits α-Amy and α-Glu activity, thereby decreasing blood sugar levels (Meng et al., 2016). CDK5 and ERS are closely related to β-cell exhaustion in T2DM. Studies have shown that myricetin can prevent induced pancreatic β-cell dysfunction by inhibiting CDK5-p66Shc signaling and ERS (Song et al., 2021).

### Kaempferol

Kaempferol is a natural product of flavonoids with antidiabetic activities in Chinese medicinal botanical drugs, such as Kaempferia galanga L. (Zingiberaceae; Kaempferiae Rhizoma). Kaempferol can increase the expression or activity of antioxidant enzymes (e.g., SOD, CAT, and heme oxygenase-1) and inhibit the activity of ROS-producing enzymes (e.g., xanthine oxidase) to express its antioxidant effect. Kaempferol (10 μM) inhibited dRib-induced intracellular ROS, apoptosis, and lipid peroxidation, and kaempferol was effective in scavenging Fenton-generated hydroxyl radicals and peroxynitrite (Lee Y. J. et al., 2010; Calderon-Montano et al., 2011). These results suggest that kaempferol protects HIT-T15 cells from dRib-induced oxidative damage. Kaempferol has a specific anti-inflammatory effect through variety of mechanisms. The activation of NF-κB and TNF-α can induce inflammation, and kaempferol exhibits anti-inflammatory activity by inhibiting the activity of NF-κB and TNF-α (Calderon-Montano et al., 2011). AP-1 (activator protein 1) is a transcriptional regulator composed of members of the Fos and Jun family involved in inflammation, and kaempferol (50 μM) has been shown to inhibit the activation of AP-1 in PC3 cells (Gopalakrishnan et al., 2006). Cyclooxygenase (COX), lipoxygenase (LOX), and inducible nitric oxide synthase (iNOS) are essential enzymes of eicosanoid synthesis in inflammation, and kaempferol exerts anti-inflammatory by inhibiting the activity of these enzymes (e.g., COX, LOX, and iNOS) (Calderon-Montano et al., 2011). Furthermore, kaempferol can inhibit key mediators of oxidative stress-induced inflammation, such as NO and iNOS (Alam et al., 2020). *In vivo* study, kaempferol (50, 150 mg/kg) reduced liver inflammation by downregulating IKK and inhibiting NF-κB pathway activation (Luo et al., 2015). Kaempferol showed significant modulation of glucose and lipid metabolism. Kaempferol stimulates glycogen synthesis in the soleus muscle through PI3K, GSK-3, MAPK, and PP1 (Cazarolli et al., 2009). In 3T3-L1 adipocytes, kaempferol (5, 10, 20, and 50 μM) may improve insulin-stimulated glucose uptake as ligands of PPARγ (Fang et al., 2008). Kaempferol may be an anti-diabetic compound protecting pancreatic beta-cell survival and function. Kaempferol (10 μM) protects β-cells by inducing cAMP generation and upregulating the protein expression of Akt and Bcl-2 (Zhang and Liu, 2011).

### Hesperidin

Hesperidin is a flavonoid mainly found in citrus plants such as the Chinese medicine Citrus × aurantium L. (Rutaceae; Citri Reticulatae Pericarpium), which exerts beneficial biological activities in treating DM. Hesperidin acts as an antioxidant by preventing the production of ROS. In a rat exhaustive exercise model, hesperidin (200 mg/kg) can prevent ROS production and avoid attenuating the activity of CAT and SOD in the chest and spleen (Estruel-Amades et al., 2019). Under high levels of glucose-induced oxidative stress and apoptosis in retinal ganglion cells (RGCs), hesperidin (12.5, 25, and 50 μmol/L) inhibited cell apoptosis by reducing the levels of Caspase-9, Caspase-3, and Bax/Bcl-2, and hesperidin also elevated the activity of enzymes related to antioxidation and recovered glutathione levels (Liu et al., 2017). Besides, hesperidin has anti-inflammatory activity. Hesperidin can regulate the NF-κB inflammatory signaling pathway by mediating the AMPK and PPAR pathways, thereby reducing inflammation and apoptosis (Xiong et al., 2019). In HFD-induced C57BL/6J mice, hesperidin (100 mg/kg) manifested systematic inflammation by suppressing the elevation of interleukin-6 (IL-6), macrophage chemoattractant protein-1 (MCP-1), and C-reactive protein (hs-CRP) (Ferreira et al., 2016). Hesperidin can treat DM by regulating glucose and lipid metabolism. Hesperidin (10, 50 μM) inhibited amylose and amylopectin digestion and significantly reduced glucose-6-phosphatase activity in HepG2 cells (Shen et al., 2012). Furthermore, hesperidin (40, 80, 120, 160, and 200 μM) blocked the enzyme entrance channel, restrained the combination between substrates and enzyme active sites, and inhibited hepatic gluconeogenesis by preventing the production of pyruvate, α-ketoglutarate, and oxaloacetate (Zareei et al., 2017). PPAR-c is a nuclear protein transcription factor closely related to lipid and glucose metabolism. In a vitro experiment, hesperidin increased glucose uptake and PPAR-γ activity or PPAR-γ mRNA expression in C2C12 myotubes. In contrast, *in vivo* experiment, hesperidin improved the impaired fasting glucose level and glucose tolerance (Shin et al., 2013). Hesperidin can regulate lipid metabolism by regulating adipokines, cytokines, and lipogenesis-related genes to some extent. In addition, hesperidin can affect IR and improve glucose homeostasis and insulin sensitivity (Xiong et al., 2019).

### Baicalein

Baicalein is one of the flavonoids with the highest content in the TCM Scutellaria baicalensis Georgi (Lamiaceae; Scutellariae Radix). Baicalein can inhibit inflammation and IR by activating AMPK in the treatment of diabetic mice. In HFD-induced mice treated with 400 mg/kg of 0.5% baicalein, a study showed that the activation of AMPK can downregulate insulin receptor substrate 1 (IRS-1) and Akt through phosphorylation and can also reduce SREBP-1c and FAS gene transcription and upregulate PPAR-α gene expression and fat target genes to inhibit cholesterol and fatty acid synthesis, thereby further achieving the purpose of treating DM (Pu et al., 2012). Baicalein (80 mg/kg) shuts down lipid metabolism by downregulating the related genes SREBP-1c and FAS, critical enzymes for cholesterol and fatty acid synthesis in HFD-fed rats, and even reduces serum cholesterol, FAA, and TNF-α by activating ACC and AMPK during their phosphorylation (Guo et al., 2009). In addition, baicalein can also delay glucose absorption by inhibiting digestive enzymes such as α-Amy and α-Glu. Therefore, baicalein can be an auxiliary drug for diabetic dietary treatment (Zhang et al., 2017). Studies have pointed out that baicalein can inhibit pancreatic β-cell damage by inhibiting the expression of 12-lipoxygenase (Deschamps et al., 2006). Another study showed that baicalein could also enhance mitochondrial function in β-cells through a cAMP-dependent pathway, thereby increasing the vitality and function of β-cells (Dinda et al., 2017).

### Apigenin

Apigenin is a flavonoid widely present in nature, especially in vegetables, such as *Apium graveolens* var. dulce (Mill.) Pers. (Umbelliferae), Petroselinum crispum (Mill.) Fuss (Umbelliferae), and Matricaria chamomilla L. (Asteraceae). According to research, apigenin shows bioactivity in diabetes, and apigenin may treat DM through different mechanisms. Apigenin exerts its antioxidant effect mainly by enhancing antioxidants and neutralizing active oxygen. For example, apigenin can prevent DM and complications by interacting with and neutralizing ROS in cells (Wang et al., 2014). In alloxan-treated mice, apigenin can protect the liver by enhancing the activity of antioxidants (such as CAT) with the administration of 0.78 mg/kg of apigenin for 10 consecutive days (Panda and Kar, 2007). Besides, apigenin can regulate lipid peroxidation in alloxan-treated mice, where the group with reduced blood lipids and blood sugar has increased SOD activity and glucose tolerance compared with the control group (Panda and Kar, 2007). Apigenin has a specific anti-inflammatory role through different pathways, including p38/MAPK and PI3K/Akt. It can also prevent IKB degradation and nuclear translocation of NF-κB and reduce COX-2 activity at the same time (Lee J.-H. et al., 2007). Other studies have shown that apigenin can protect β-cell function by inhibiting inflammatory signaling pathways (Bai et al., 2019).

## Terpenoids

Terpenoids are compounds whose molecular skeleton takes isoprene units as essential structural cores. The number of isoprene units can be divided into monoterpenes, sesquiterpenes, and diterpenes. Monoterpenes and sesquiterpenes are the main components of volatile plant oils. The terpenoids involved in this article include catalpol, login, oleanolic acid, and crocin (Table 4). Terpenoids are widely distributed in nature and are the most abundant in angiosperms, which have functions such as antioxidant, anti-inflammatory, regulation of glucose metabolism, and protection of pancreatic islets. First, terpenoids can exert their antioxidant effect by increasing the antioxidant enzymes in the body, inhibiting ROS generation, and scavenging free radicals. For example, crocin can achieve its antioxidant effect by scavenging free radicals. (Korani et al., 2019). Second, terpenoids can exert anti-inflammatory effects by inhibiting proinflammatory cytokines, such as oleanolic acid, by reducing the serum levels and gene expression of the proinflammatory cytokines IL-1β, IL-6, and TNF-α. Third, terpenoids can regulate glucose metabolism by inhibiting α-Glu and regulating PPARγ, such as catalpol and oleanolic acid.

**TABLE 4:** Representative antidiabetic terpenoids.

Bioactive metabolites	Detail	Cell lines/model	Dose	Negative/positive control	Duration	References
Catalpol	Increased the level of antioxidant enzymes, reduced MDA and NOX4 protein	HFD/STZ-induced mice	100, 200 mg/kg	—	4 weeks	Yan et al. (2018)
	Improved the function of hepatic mitochondria by activating Mfn1 and downregulating Fis 1 and Drp 1	HFD/STZ-induced mice	50, 100, and 200 mg/kg	PC: Metformin (200 mg/kg)	—	Xu et al. (2015)
	Ameliorated IR and inflammation by suppressing the JNK and NF-kB pathways	HFD-induced mice	100 mg/kg	—	16 weeks	Zhou et al. (2015)
	Inhibited the expression of pro-inflammatory mediates, phosphorylation of MAPK, production of ROS, degradation of IκBα, and the nuclear localization of NF-κB to exert an anti-inflammatory effect	THP-1 cells	100, 300, and 500 μM	—	30 min	Choi et al. (2013)
	Modulated glucose metabolism by ameliorate gene expression especially SOCS3	db/db mice	25, 50, 100, and 200 mg/kg	PC: Metformin	56 days	Liu et al. (2018)
	Activated the PI3K/AKT pathway to promote glycogen production and inhibit gluconeogenesis	HepG2 cells	20, 40, and 80 μM	—	24 h	Yan et al. (2018)
	Activated the PI3K/AKT pathway	STZ/HFD-induced diabetic rats	100, 200 mg/kg	—	4 weeks	
	Improved insulin sensitivity and mitochondrial respiration through the insulin signaling pathway and AMPK/SIRT1/PGC-1α/PPAR-γ activation	STZ/HFD-induced diabetic mice	100, 200 mg/kg	PC: Metformin (200 mg/kg)	4 weeks	Yap et al. (2020)
Loganin	Inhibited the production of ROS and the activation of NLRP3 inflammasome	RSC96 cells	0.1, 1, 10, 25, and 50 μM	—	2 h	Cheng et al. (2020)
Oleanolic acid	Increased the activity of antioxidant enzymes, reduced MDA levels	alloxan-induced diabetic rats	60, 100 mg/kg	—	40 days	Gao et al. (2009)
	Decreased level of IL-1 β, IL-6, and TNFα	db/db mice	20 mg/kg	NC: 2% Tween 80 in sterile saline	2 weeks	Wang et al. (2013)
	Ameliorated IR by the PPARγ signaling through the upregulation of hepatocyte nuclear factor 1b (HNF1b)	Aroclor 1254-induced mice	50 mg/kg	PC: Vitamin C (100 mg/kg)	10 weeks	Su et al. (2018)
Crocin	Decreased MDA levels and increased GSH-px and SOD activities	Male C57BL/6J mice	5, 10, and 20 mg/kg	—	21 days	Zheng et al. (2007)
	Attenuated Tumor Necrosis Factor-alpha (TNF-α) and interleukin-6 (IL-6) to exert an anti-inflammatory function	STZ-induced diabetic rats	10, 20, and 30 mg/kg	NC: physiological saline	4 weeks	Samarghandian et al. (2016)

### Catalpol

Catalpol is a terpenoid extracted from the TCM Rehmannia glutinosa (Gaertn.) DC. (Orobanchaceae; Rehmanniae Radix), which has been demonstrated to have antidiabetic properties. Catalpol plays a vital role in oxidative stress. In HFD/STZ-induced mice, catalpol (100, 200 mg/kg) increased GSH and SOD, reduced MDA levels, and decreased NOX4 protein overexpression (Yan et al., 2018). In DM mice and GC-2 cells, catalpol prevented ROS production by inhibiting the expression of RAGE, Nox4, and NF-κB p65, thereby reducing the oxidative stress induced by AGEs (Jiao et al., 2020). In addition, catalpol can reduce oxidative stress by increasing the formation of mitochondria. Studies have pointed out that catalpol (50, 100, 200 mg/kg) can improve the function of hepatic mitochondria by activating Mfn1 and downregulating Fis one and Drop one in HFD/STZ-induced mice (Xu et al., 2015; Bhattamisra et al., 2021). Studies have indicated that catalpol reduced inflammation by suppressing ROS production, pro-inflammatory factors, and the NF-κB pathway. In HFD-induced mice, catalpol (100 mg/kg) exerted anti-inflammatory effects by inhibiting M1 proinflammatory factors and increasing M2 anti-inflammatory factors in adipose tissue, and catalpol ameliorated IR and inflammation by suppressing the JNK and NF-kB pathways (Zhou et al., 2015). In THP-1 cells, catalpol (100, 300, and 500 μM) inhibited the expression of proinflammatory mediators (e.g., MCP-1, TNF-α, iNOS), phosphorylation of MAPK, production of ROS, degradation of IκBα and the nuclear localization of NF-κB (Choi et al., 2013). Glucose and lipid metabolism are also important in DM treatment. Catalpol can prevent gluconeogenesis by activating AMPK and inhibiting the expression of PEPCK and G6Pase proteins (Bhattamisra et al., 2021). In HepG2 cells, catalpol (20, 40, and 80 μM) activated the PI3K/AKT pathway to promote glycogen production and inhibit gluconeogenesis (Yan et al., 2018). Catalpol (25, 50, 100, and 200 mg/kg) can remarkably ameliorate gene expression, especially SOCS3, to modulate glucose metabolism in db/db mice (Liu et al., 2018). Furthermore, catalpol benefits IR and insulin sensitivity. Both *in vivo* and *in vitro* studies suggest catalpol can ameliorate IR; for example, catalpol may suppress IR by inhibiting the overexpression of NOX4 and activating AMPK in glucosamine-induced HepG2 cells (Yan et al., 2018). In addition, catalpol (100, 200 mg/kg) can improve insulin sensitivity *via* the insulin-related signaling pathway and AMPK/SIRT1/PGC-1α/PPAR-γ activation in STZ/HFD-induced diabetic mice (Yap et al., 2020).

### Oleanolic acid

Oleanolic acid is a terpenoid widely present in plants, which can be extracted from the TCM Ligustrum lucidum W.T.Aiton (Oleaceae; Ligustri Lucidi Fructus). It has been demonstrated that oleanolic acid possesses antidiabetic effects in treating DM. Oleanolic acid may increase the activities of antioxidant enzymes to exert antioxidant effects. In alloxan-induced diabetic rats, oleanolic acid (60, 100 mg/kg) reduced the MDA level and increased the activities of SOD and GSH-Px, which indicated that oleanolic acid possessed antioxidant effects (Gao et al., 2009). Furthermore, studies suggest that oleanolic acid elevated antioxidant enzymes (e.g., SOD, CAT, and GSH-Px) *via* the transcription factor Nrf2 (Castellano et al., 2013). Oleanolic acid also has anti-inflammatory effects. *In vivo* and *in vitro* studies indicated that oleanolic acid possesses anti-inflammatory properties, mediated by decreasing the NF-κB expression and TNF-α generation (Lee et al., 2013). In db/db mice, oleanolic acid (20 mg/kg) ameliorates the inflammatory condition by downregulating of IL-1 β, IL-6, and TNF-α in the circulation, which improves hepatic IR (Wang et al., 2013). Oleanolic acid has a specific effect on regulating glucose and lipid metabolism. The experiments showed that oleanolic acid could control blood sugar by inhibiting α-Glu (Ding et al., 2018). PPARs are a family of nuclear transcription factors of the sterol receptor superfamily, which can regulate lipid and glucose metabolism (Berger et al., 2005). *In vitro* studies, oleanolic acid stimulated GLUT-4 translocation in C2C12 myoblasts, inhibited lipid accumulation in 3T3-L1 adipocytes, and affected PPARγ/α. These results suggested that oleanolic acid may have an antihyperglycemic effect (Loza-Rodríguez et al., 2020). Subsequently, oleanolic acid (25 mg/kg) improved IR through the IRS-1/PI3K/Ak pathway (Li et al., 2014). In addition, oleanolic acid (50 mg/kg) could ameliorate IR by the PPARγ signaling through the upregulation of hepatocyte nuclear factor 1b (HNF1b) and in aroclor 1254-induced mice (Su et al., 2018).

### Crocin

Crocin, an uncommon terpenoid, is one of the main components of the TCM Crocus sativus L. (Iridaceae; Croci Stigma), which can treat DM and its complications through its bioactivities. MDA and ER are closely related to cellular oxidative stress. Experiments indicated that crocin can exert its antioxidant effect by scavenging free radicals, reducing MDA levels, increasing GPx and SOD activities, and regulating mRNA expression of ER stressors (Korani et al., 2019). Crocin (5, 10, and 20 mg/kg) decreased MDA levels and increased GSH-px and SOD activities in the reperfusion-induced oxidative/nitrative injury mice, thereby expressing its antioxidant effect (Zheng et al., 2007). Meanwhile, crocin has an anti-inflammatory effect. Crocin (10, 20, and 30 mg/kg) inhibited the activation of inflammatory signaling by suppressing the mRNA expression of proinflammatory cytokines (e.g., TNF-α, IL-6) in STZ-induced diabetic rats (Samarghandian et al., 2016).

### Loganin

Loganin, a common terpenoid, is one of the main components of Cornus officinalis Siebold and Zucc. (Cornaceae; Corni Fructus) has bioactivity in DM and its complications. The experiment of RSC96 Schwann cells pretreated with loganin (0.1, 1, 10, 25, and 50 µM) indicated that the antioxidant effects of loganin prevented the apoptosis of RSC96 Schwann cells by inhibiting the production of ROS and the activation of NLRP3 inflammasomes, thereby playing a role in neuropathy caused by DM (Cheng et al., 2020).

## Alkaloids

Alkaloids are a class of compounds with complex structures. Compared with other natural products, alkaloids have the following characteristics: 1) the structure contains at least one nitrogen atom; 2) nitrogen atoms are derived from amino acids or purine nucleus or amination of steroids and terpenes; 3) alkaloids are basic or neutral; 4) alkaloid generally do not include peptides with a molecular weight greater than 1,500. The alkaloids involved in this article are berberine, matrine, and betaine (Table 5). Alkaloids are mainly derived from natural plants and have physiological activities such as antioxidant, anti-inflammatory, regulation of glucose metabolism, and antibacterial activities. Alkaloids can exert their antioxidant effects by reducing oxidative stress and increasing the expression of antioxidant enzymes. They exert their anti-inflammatory effects by inhibiting NF-κB and regulating glucose metabolism by activating AMPK. For example, betaine can improve oxidative stress, inhibit NF-κB, and activate AMPK (Jung G. Y. et al., 2013).

**TABLE 5:** Representative antidiabetic alkaloids.

Bioactive metabolites	Detail	Cell lines/model	Dose	Negative/positive control	Duration	References
Berberine	Increased the expression of antioxidant enzyme mRNA, increased the content of antioxidant enzymes	STZ/HFD-induced diabetic rats	75, 150, and 300 mg/kg	—	16 weeks	Zhou and Zhou, (2011)
	Possessed antioxidant efffect *via* a PI3K/Akt-dependent manner	NSC34 neuronal cells	0.1–10 nM	—	24 h	Hsu et al. (2012)
	Exerted anti-inflammatory *via* AMPK and Nrf2 pathways	RAW 264.7 cells	5 μM	—	2 h	Mo et al. (2014)
		LPS-induced mice	10 mg/kg	—	6 h	
	Reduced transcription factors and inhibit gluconeogenesis	STZ/HFD-induced diabetic rats	380 mg/kg	NC: PBS	5 weeks	Xia et al. (2011)
	Inhibited hepatic gluconeogenesis by upregulating the mRNA and protein expression of HNF-4α	Rat pancreatic islets	1, 3, 10, and 30 μM	PC: Glibenclamide (1 μM)	24 h	Wang et al. (2008)
	Reduced of IR through the phosphorylation of InsR and its IRS-1	db/db mice	100 mg/kg	PC: Rosiglitazone (10 mg/kg)	2 weeks	Chen et al. (2010)
	Improved IR *via* stimulation of AMPK activity	H9c2 cells	3.125–100 μM	—	30 min, 12 h	Chang et al. (2013)
	Increased the expression of insulin receptor substrate 2 mRNA	NAFLD rats	187.5 mg/kg	PC: Pioglitazone (10 mg/kg)	4 weeks	Xing et al. (2011)
	Stimulated GLP-1 secretion to promote insulin secretion and improve β-cell function	STZ-induced diabetic rats	120 mg/kg	—	5 weeks	Yu et al. (2010)
Betaine	Reduced endoplasmic reticulum and oxidative stress	db/db mice	1 g betaine/100 g diet	—	—	Jung G. Y et al. (2013)
	Inhibited activation of NF-κB	SD Rats	30, 60, and 120 mg/kg	—	10 days	Go et al. (2005)
	Increased activated AMPK	High-sucrose diet-fed rats	1% (wt/vol)	—	16 weeks	Song et al. (2007)
Matrine	Elevated NO levels and eNOS activity, and upregulated ROS production	Ox-LDL-induced HUVECs	5, 20, and 80 μM	—	12 h	Zhang et al. (2019)
	Reduced TNF-α and IL-6, while elevating IL-10 levels	HFD-induced diabetic mice	0.5, 2.5, and 10 mg/kg	—	12 weeks	
	Ameliorated glucose and lipid metabolism through the suppression of ER stress and regulation of PK2/PKRs pathway	STZ/HFD-induced diabetic mice	40 mg/kg	—	8 weeks	Zhang et al. (2022)
	Elevated GLP-1 protein expression	STZ/HFD -induced diabetic mice	0.5, 2.5, and 10 mg/kg	—	6 weeks	Zhang et al. (2019)

### Berberine

Berberine is a quaternary ammonium alkaloid that is mainly extracted from the TCM Coptis chinensis Franch. (Ranunculaceae; Coptidis Rhizoma), which has various biological mechanisms in treating DM. Studies have demonstrated that berberine has antioxidant effects through different mechanisms. In STZ/HFD-induced diabetic rats, berberine (75, 150, and 300 mg/kg) decreased MDA levels and increased the activities of antioxidant enzymes (e.g., SOD, GSH, GSH-Px, CAT) in rat liver cells, thereby overcoming oxidative stress (Zhou and Zhou, 2011). Nrf2 can activate the expression of antioxidant enzymes (e.g., SOD, and GSH), and studies have shown that berberine (0.1–10 nM) can activate Nrf2 nuclear translocation and protect against oxidative damage *via* a PI3K/Akt-dependent pathway in NSC34 neuronal cells (Hsu et al., 2012). Proinflammatory cytokines (e.g., TNF-α, IL-6, iNOS, and COX2) are closely related to DM, and NF-κB plays an important role in producing proinflammatory cytokines. Berberine can inhibit NF-κB through the suppression of phosphorylation of IκB kinase-β (IKK-β) and RhoA/ROCK signaling pathways (Chang et al., 2015). Berberine exerts anti-inflammatory effects *via* the AMPK and Nrf2 pathways *in vivo* and *in vitro*. In RAW264.7 cells (treated with 5 μmol/L berberine) and LPS-induced mice (treated with 10 mg/kg berberine), the expression of inflammatory genes (e.g., iNOS, COX2, and IL-6) and the generation of NO and ROS were attenuated, but Nrf2-targeted antioxidative genes increased (Mo et al., 2014). In STZ/HFD-induced diabetic rats, berberine (380 mg/kg) mainly regulated glucose and lipid metabolism by reducing the expression of nuclear transcription factors (e.g., FoxO1, SREBP1, and ChREBP) in the liver (Xia et al., 2011). Hepatic nuclear factor 4α (HNF-4α) is a factor of the hepatic nuclear family that can inhibit gluconeogenesis. Berberine (1, 3, 10, and 30 μmol/L) can inhibit hepatic gluconeogenesis by upregulating the mRNA and protein expression of HNF-4α in rat pancreatic islets (Wang et al., 2008). PPARs (peroxisome proliferator-activated receptors) play an important role in regulating target gene expression related to glucose and lipid metabolism, including PPARγ, PPARα, and PPARδ (Berger and Moller, 2002). Berberine can upregulate hepatic low-density lipoprotein receptor (LDLR) by activating the c-Jun N-terminal kinase (JNK) pathway in HepG2 and HEK-293 cells (Lee S. et al., 2007). Studies have identified that berberine can affect insulin functions and β-cells. The insulin receptor (InsR) is a cell membrane glycoprotein essential in IR. In human liver cells and rat models, berberine increased the InsR mRNA and protein expression to reduce IR (Kong et al., 2009). Moreover, another study pointed out that berberine (100 mg/kg) affected the phosphorylation of InsR and its insulin receptor substrate-1 (IRS-1) in db/db mice, which ultimately led to reduction in IR (Chen et al., 2010). In H9c2 cells, the study indicated that berberine (3.125–100 μmol/L) improved IR at least *via* the stimulation of AMPK activity, and berberine also increased glucose consumption and glucose uptake (Chang et al., 2013). Berberine (5 mg/kg) improved insulin-sensitizing and lipid-lowering properties in db/db mice, and berberine activated the AMPK activity in 3T3-L1 cells and stimulated GLUT4 translocation in L6 myotubes (Lee et al., 2006). Moreover, berberine (187.5 mg/kg) enabled the upregulation of insulin receptor substrate 2 mRNA in nonalcoholic fatty liver disease (NAFLD) rat liver (Xing et al., 2011). Glucagon-like peptide-1 (GLP-1) is an important incretin that promotes insulin secretion and inhibits glucagon secretion. GLP-1 receptors play an important role in the survival of pancreatic islet cells. Berberine (120 mg/kg) may significantly stimulate GLP-1 secretion to promote insulin secretion and improve β-cell function in STZ-induced diabetic rats (Yu et al., 2010).

### Matrine

Matrine is an alkaloid extracted in the Chinese medicine *Sophora flavescens* Aiton (Fabaceae; Sophorae Flavescentis Radix), which has beneficial effects on DM treatment. Studies have pointed out that marine exerts antioxidant effects by reducing ROS production *via* the PI3K/Akt pathway. Matrine (5, 20, and 80 μmol/L) could elevate NO levels and eNOS activity and downregulate intracellular ROS production in ox-LDL-induced HUVEC injury, mediated through the PI3K/Akt pathway (Zhang et al., 2019). Furthermore, matrine could block inflammatory factors to express their anti-inflammatory effect. Matrine (0.5, 2.5, and 10 mg/kg) reduced TNF-α, IL-6, and elevated IL-10 levels, demonstrating that marine had anti-inflammatory effects by regulating inflammatory cytokines in HFD-induced mice (Zhang et al., 2019). In addition, the matrine significantly affects glucose and lipid metabolism through ER stress and the PK2/PCRs pathway. In STZ/HFD-induced mice, matrine (40 mg/kg) reduced FBG, improved glucose tolerance, suppressed lipid production while preventing ER stress-related protein overexpression, and increased PK2, PKR1, and PKR2 expression. These results indicated that matrine ameliorated glucose and lipid metabolism by suppressing ER stress and regulating the PK2/PCRs pathway (Zhang et al., 2022). GLP-1 is a hormone secreted by the intestines that can regulate glucose metabolism by stimulating insulin secretion, and studies have pointed out that matrine can stimulate GLP-1 secretion and improve IR. A dose of matrine treatment (10 mg/kg) elevated GLP-1 protein expression and plasma GLP-1 levels stimulated by CaSR, thus improving glucose homeostasis in STZ/HFD-induced mice (Guo et al., 2021).

### Betaine

Betaine, a quaternary ammonium alkaloid, is widely found in plants such as the Chinese medicine Lycium barbarum L. (Solanaceae; Lycii Fructus), which can treat DM through its activities. Studies have pointed out that the primary antioxidant mechanism of betaine may be achieved by improving the metabolism of SAA, and reducing the endoplasmic reticulum and oxidative stress in db/db mice (Jung G. Y. et al., 2013). In addition, researchers have found that betaine has protective effects attributed to antioxidant defense by ameliorating impaired sulfur amino acid metabolism in alcoholic liver injury (Jung Y. S. et al., 2013). Betaine is a valuable agent for suppressing inflammation by inhibiting the NF-κB and IL-1β. Studies have indicated that betaine (30, 60, and 120 mg/kg) inhibits NF-κB activation *via* NIK/IKK and MAPKs to suppress the production of proinflammatory cytokines in old SD rats (Go et al., 2005). Additionally, betaine can inhibit the NLRP3 inflammasome by enhancing IRS activity to activate PKB/Akt pathway (Zhao et al., 2018). In addition, betaine exerts anti-inflammatory effects by restoring energy metabolism and reducing endoplasmic reticulum stress and apoptosis. In high-sucrose diet-induced mice, betaine (1% wt/vol) regulated glucose and lipid metabolism by activating AMPK and other lipid metabolism-related factors (Song et al., 2007). Both *in vivo* and *in vitro* studies, betaine improved IR by increasing IRS1 phosphorylation and ameliorating downstream pathways related to gluconeogenesis and glycogen synthesis *via* the PI3K/Akt signaling pathway (Kathirvel et al., 2010).

## Other bioactive metabolites

In addition to flavonoids, terpenes, and alkaloids, other types of active ingredients in TCM also play an essential role in diabetes. After a literature search, it was found that compounds such as polyphenols, phenylpropanoids, and quinones also have antidiabetic properties, such as tangerine, curcumin, resveratrol, emodin, chrysophanol, and cryptotanshinone. Therefore, the following representative active ingredients are selected for elaboration (Table 6).

**TABLE 6:** The Other Representative Antidiabetic Bioactive metabolites.

Bioactive metabolites	Detail	Cell lines/model	Dose	Negative/positive control	Duration	References
Curcumin	Increased activities of antioxidant enzymes	STZ-induced diabetic mice	10 mM	—	28 days	El-Azab et al. (2011)
	Attenuated oxidative stress through Sirt1-Foxo1 and PI3K-Akt signaling pathways	HFD-induced diabetic rats	100 mg/kg	—	12 weeks	Ren et al. (2020)
	Reduced TNF-α level	HFD-induced diabetic rats	80 mg/kg	PC: Rosiglitazone (1 mg/kg)	60, 75 days	El-Moselhy et al. (2011)
	Decreased NF-κB expression, activated AMPK expression, and reversed the reduction of PPARγ expression	db/db mice	0.75% v/v	—	8 weeks	Jiménez-Flores et al. (2014)
	Prevented the membrane translocation of GLUT2 through the interruption of the p38 MAPK signaling pathway	Hepatic stellate cells (HSCs)	0–30 μM	PC: Rosiglitazone (0–10 μM)	24 h	Lin and Chen, (2011)
	Increased hepatic GK activity, inhibited hepatic gluconeogenic enzyme activities	db/db mice	0.02% (wt/wt)	—	6 weeks	Seo et al. (2008)
	Reduced G6Pase expression and activity by activating AMPK	Gestational diabetes mouse model	100 mg/kg	NC: 0.1% DMSO in saline	20 days	Lu et al. (2019)
	Ameliorated IR by reducing plasma peptide levels of IAPP and GSK-3β	Adults	180 mg/day	—	12 weeks	Thota et al. (2020)
Cinnamaldehyde	Elevated antioxidant enzymes activities	STZ-induced diabetic rats	5, 10, and 20 mg/kg	PC: Glibenclamide (0.6 mg/kg)	45 days	Subash-Babu et al. (2014)
	Attenuated oxidative stress through the activation of Nrf2 pathway	db/db mice	0.02% v/v	—	12 weeks	Wang et al. (2020)
	Regulated glucose/lipid metabolism through RBP4-GLUT4 system	HFD-induced diabetic rats	40 mg/kg	—	8 weeks	Zhang et al. (2008)
	Ameliorated IR by elevating PPARγ gene expression	FSD/STZ-induced rats	20 mg/kg	—	1 week	Hosni et al. (2017)
Tan IIA	Reduced the level of MDA, increase dthe activity of SOD; suppressed endoplasmic reticulum (ER) stress activation	STZ-induced diabetic rat	2, 4 mg/kg	—	6 weeks	Chen J et al. (2018)
	Decreased inflammatory cytokines	STZ/HFD-induced diabetic rats	10 mg/kg	NC: PBS (10 mg/kg)	8 weeks	Yuan et al. (2018)
	Increased peroxisome proliferator-activated receptor γ (PPARγ) and its target genes	HFD-induced diabetic rats	crude 0.4 g/kg	PC: Rosiglitazone	6 weeks	Xie et al. (2015)

### Curcumin

Curcumin, a polyphenolic compound, possesses different mechanisms in treating DM, which can be extracted from the traditional Chinese Curcuma longa L. (Zingiberaceae; Curcumae Longae Rhizoma). According to research, curcumin acts as an antioxidant agent by enhancing the activity of antioxidant enzymes and scavenging radicals. Despite the presence or absence of bone marrow transplantation, curcumin (10 mM) blocked the MDA level and increased the activity of antioxidant enzymes in STZ-induced mice, alleviating lipid peroxidation (El-Azab et al., 2011). In STZ/HFD-induced rats, curcumin (100 mg/kg) attenuated oxidative stress and suppressed diabetic cardiomyopathy apoptosis through Sirt1-Foxo1 and PI3K-Akt signaling pathways (Ren et al., 2020). The anti-inflammatory effect of curcumin contributes to the release of inflammatory cytokines and decreases the NF-κB pathway. NF-κB is a critical transcription factor in the inflammatory response, while AMPK and PPARγ play a role in glucose/lipid metabolism. Curcumin (0.75% v/v) decreased NF-κB expression, activated AMPK expression, and reversed the reduction in PPARγ expression in db/db mice (Jiménez-Flores et al., 2014). Glucose and lipid metabolism are essential in DM, and curcumin can regulate glucose and lipid metabolism through glucose transporters, AMPK, glucose, and lipid metabolism-related enzymes (e.g., GK, ACAT, HMG-CoA reductase). Glucose transporter 2 (GLUT2) is a transmembrane transporter that promotes glucose uptake, and curcumin blocks the translocation of GLUT2. Curcumin (0–30 μM) prevented the membrane translocation of GLUT2 by interrupting the p38 MAPK signaling pathway. It inhibited the gene expression of GLUT2 through the stimulation of PPAγ activity and the decrease in oxidative stress, thus contributing to the elimination of hyperglycemia-stimulated HSC activation, which may have beneficial effects on glucose metabolism (Lin and Chen, 2011). AMPK is a central lipid and glucose metabolism regulator, stimulating muscle glucose uptake. High-dose curcumin (100 mg/kg) reduced the expression and activity of the key enzymes in gluconeogenesis (G6Pase) by activating AMPK in a gestational diabetes mouse model (Lu et al., 2019). Glucose and lipid metabolism-related enzymes also play an important role in glucose and lipid metabolism. In db/db mice, curcumin (0.02%, wt/wt) increased hepatic GK activity, inhibited hepatic gluconeogenic enzyme activities (e.g., G6Pase, PEPCK), and reduced lipid regulating enzyme activities (e.g., HMG- CoA reductase, ACAT) and lipid profiles (e.g., plasma fatty acids, total cholesterol, and TGs) (Seo et al., 2008). Thus, it has been proven that curcumin regulates glycose and lipids. In addition, curcumin could ameliorate IR by downregulating IAPP, GSK-3β, and TNF-α. Islet amyloid polypeptide (IAPP) and GSK-3β are critical peptides in IR. Supplementation with curcumin (180 mg/day) reduced plasma peptide levels of IAPP and GSK-3β in adults with a high risk of type 2 diabetes, thus ameliorating IR (Thota et al., 2020). Curcumin (80 mg/kg) suppressed TNF-α levels and free fatty acid levels in HFD-induced rats and improved insulin sensitivity due to its anti-inflammatory effect by suppressing TNF-α levels (El-Moselhy et al., 2011).

### Cinnamaldehyde

Cinnamaldehyde, a phenylpropanoid compound, has rich activities against DM, which can be extracted from the TCM Neolitsea cassia (L.) Kosterm. (Lauraceae; Cinnamomi Cortex). Studies have pointed out that cinnamaldehyde could have antioxidant effects by elevating antioxidant enzyme activity and activating the Nrf2 pathway. Cinnamaldehyde (20 mg/kg) elevated SOD, GSH, GPx, and CAT, and reduced lipid peroxidation-related TBARS in the pancreatic β-cell injury of STZ-induced diabetic rats. Thus, cinnamaldehyde (5, 10, and 20 mg/kg) protected pancreatic β-cell injury *via* the elevation of antioxidant enzyme activities (Subash-Babu et al., 2014). In db/db mice, cinnamaldehyde reduced ROS levels, preserved NO production, and elevated Nrf2 and its targeted genes (such as HO-1 and NQO-1), and these results suggested that cinnamaldehyde (0.02% v/v) attenuated oxidative stress through the activation of the Nrf2 pathway (Wang et al., 2020). Cinnamaldehyde also has anti-inflammatory effects by inhibiting inflammatory factors and NF-κB activity. Several studies have demonstrated that cinnamaldehyde regulates glucose/lipid metabolism through GLUT4. RBP4 and GKUT4 are closely related and important factors in glucose and lipid metabolism. Cinnamaldehyde (40 mg/kg) decreased FBG and LDL levels while reducing serum RBP4 levels and elevating GLUT4 protein expression in HFD-induced rats (Zhang et al., 2008). PPARγ is a critical factor in glucose and lipid metabolism, enhancing insulin sensitivity (Feige et al., 2006). Cinnamaldehyde (20 mg/kg) elevated PPARγ gene expression and insulin levels, thus ameliorating IR in FSD/STZ-induced rats (Hosni et al., 2017).

### Tan IIA

Tan IIA is a quinone compound extracted from the TCM Salvia miltiorrhiza Bunge (Lamiaceae; Salviae miltiorrhizae radix et rhizoma), which possesses bioactivities in treating DM. Tan IIA can protect cells by regulating the level of antioxidant enzymes and reducing the production of ROS (Ansari et al., 2021). In STZ-induced diabetic rats, Tan IIA (2, 4 mg/kg) reduced MDA content and GRP78 and CHOP expression by inducing SOD activity. It also decreased neuronal apoptosis and ameliorated learning and memory by inhibiting ER stress activation (Chen J. et al., 2018). Tan-IIA has been used to treat inflammation in Chinese medicine. Due to the correlation between inflammation and diabetes, Tan-IIA can treat DM through anti-inflammatory effects (Guo et al., 2020). In STZ/HFD-induced diabetic rats, Tan IIA (10 mg/kg) may suppress the levels of inflammatory cytokines (e.g., IL-6, IL-8, and TNF-α), attenuate the level of NF-κB, and increase 5’ adenosine monophosphate-activated protein kinase levels, then overcome the symptoms of type 2 diabetes (Yuan et al., 2018). In STZ-induced diabetic rats, Tan IIA (10 mg/kg) improved renal damage by reducing oxidative stress and inflammation (Chen X. et al., 2017). In addition, studies have pointed out that Tan IIA (0.4 g crude danshen/kg) regulated glucose metabolism by increasing PPARγ and its target genes in HFD-induced diabetic rats (Xie et al., 2015).

## Discussion and outlook

DM, a chronic metabolic noncommunicable disease, has attained epidemic proportions worldwide and the existing oral drugs for DM include biguanides, sulfonylureas, α-Glu inhibitors, etc. Among them, metformin can inhibit hepatic gluconeogenesis and promote the uptake and utilization of glucose by peripheral tissues, thereby improving the body’s insulin sensitivity. Sulfonylureas can stimulate islet β-cells to secrete insulin, thereby reducing blood sugar. α-Glu inhibitors can inhibit the activity of α-Glu in the small intestine and delay the absorption of intestinal glucose. However, side effects such as hypoglycemia and gastrointestinal reactions limit these drugs’ application in the clinic. It has been known that a considerable part of the natural compounds in TCM show promising effects in treating DM. These antidiabetic components often have multiple mechanisms to improve pathological conditions and lower blood sugar through different pathways, with relatively few side effects, showing great potential for developing new drugs for treating DM. These antidiabetic molecules derived from TCM may potentially be the next generation of biguanides, sulfonylureas, α-Glu inhibitors, and GLP-1 receptor agonists.

**FIGURE 3:**
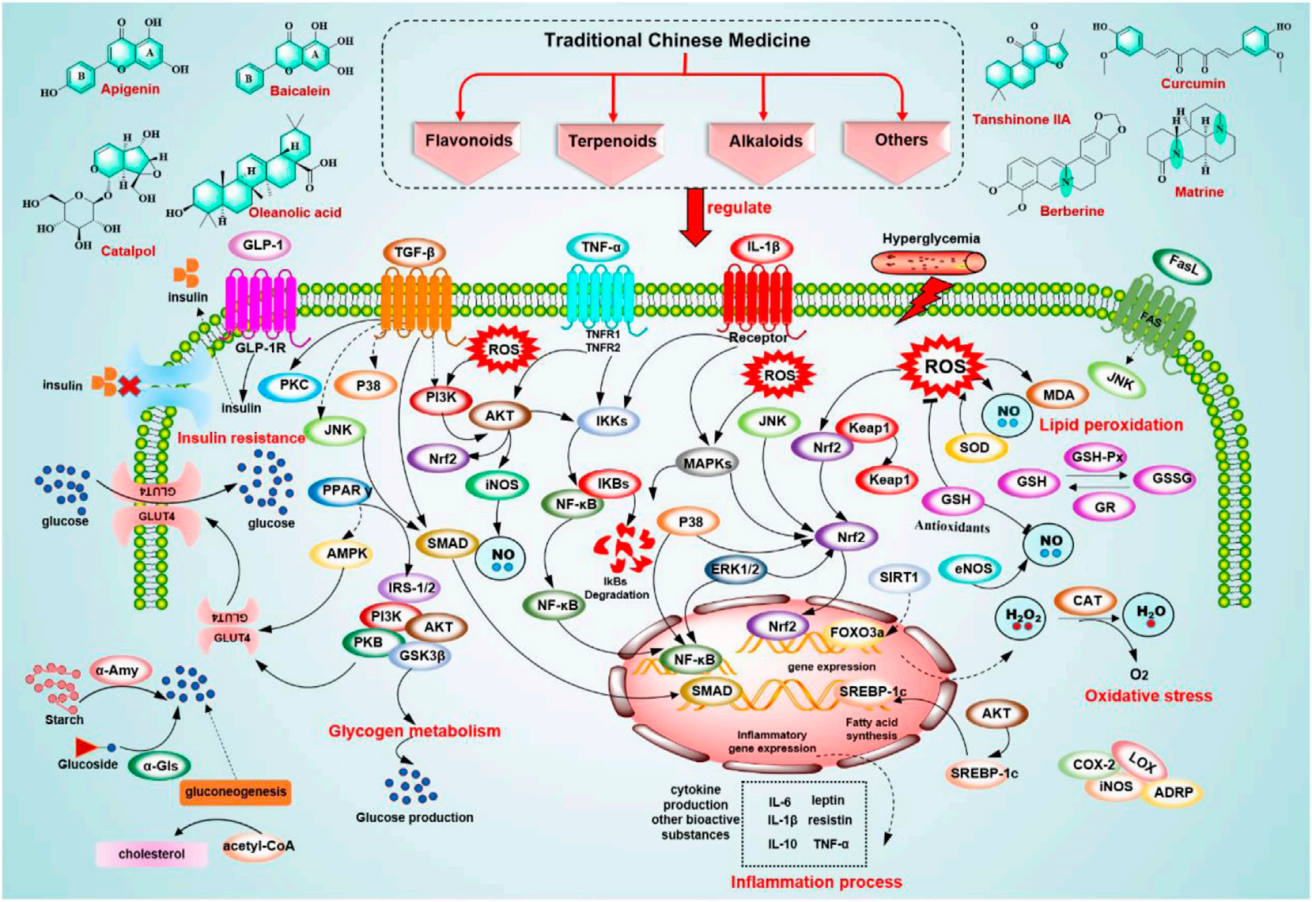
Signaling pathways involved in the treatment of T2DM of potential bioactive metabolites from TCM.

**FIGURE 4:**
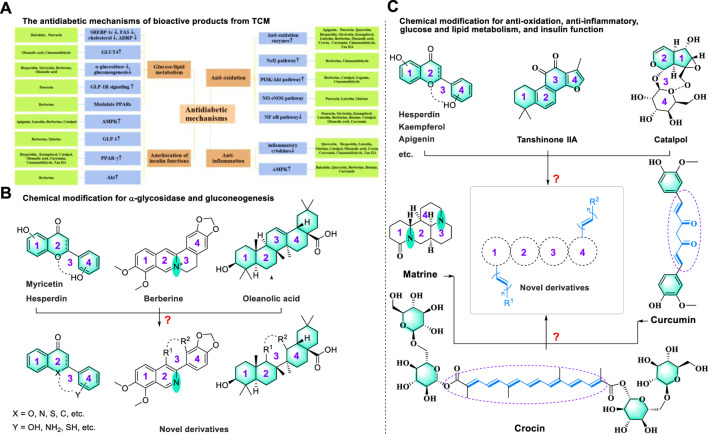
**(A)** The mechanisms of potential bioactive metabolites from TCM in the treatment of T2DM and **(B,C)** potential chemical modification for discovering novel antidiabetic drugs.

This review mainly introduces the role of flavonoids, alkaloids, terpenoids, and others from TCM in treating DM. Among them, flavonoids accounted for the majority, and alkaloids and terpenes accounted for a small proportion. Through *in vivo* and *in vitro* studies, it has been found that these bioactive metabolites treat DM mainly through antioxidation, anti-inflammation, regulation of glucose and lipid metabolism, and restoration of pancreatic islet cell function. The antioxidant effect of these components plays a significant role in the treatment of DM, mainly through the following aspects to achieve its antioxidant effect: 1) enhancing the activity of antioxidant enzymes; 2) inhibiting the production of ROS and reducing the content of MDA; and 3) scavenging free radicals in the human body. Studies have pointed out that DM is a chronic inflammatory state. The anti-inflammatory effect is mainly achieved by inhibiting the NF-κB pathway, COX, and LOX, activating AMPK, and inhibiting the production of pro-inflammatory cytokines. These natural components show potency to regulate glucose and lipid metabolism by activating AMPK, inhibiting α-Amy and α-Glu activity, and regulating the PPAR signaling pathway. Additionally, these natural compounds can promote the recovery of pancreatic islet cell function by inhibiting inflammatory signaling pathway to protect β-cell function, regulating AMPK activity and related gene expression to enhance peripheral insulin sensitivity and improve IR, and regulating the PI3K/Akt signaling pathway to protect islet cells. In brief, flavonoids, terpenoids, alkaloids, and other compounds display a multifaceted antidiabetic mechanism. Flavonoids can reduce blood glucose mainly by regulating AMPK activity, regulating PPARγ, and inhibiting α-Glu activity. For example, hesperidin can inhibit α-Glu activity and starch digestion and reduce blood glucose concentrations. The representative terpenoids catalpol and oleanolic acid regulate glucose metabolism by inhibiting α-Glu and regulating PPARγ. Interestingly, alkaloids generally play an anti-inflammatory role in DM by inhibiting NF-κB and activating AMPK to regulate glucose metabolism. For example, betaine can exert anti-inflammatory effects by inhibiting NF-κB and activating AMPK. Research on these mechanisms has provided a specific theoretical basis for DM and revealed the broad application prospects of bioactive compounds from TCM in DMs (Figures 3, 4A).

Interestingly, we noted that these four types of bioactive metabolites show similarities in the therapeutic mechanism of diabetes (Figure 4A). In particular, we found that bioactive metabolites, such as hesperidin, myricetin, berberine, and oleanolic acid, could affect glucose and lipid metabolism by downregulating α-glycosidase and gluconeogenesis to achieve a therapeutic effect on diabetes. This phenomenon may be explained by the formation of a similar transient conformation of the polycyclic skeleton. According to the medicinal chemistry principles for drug design, we proposed that the third ring moiety may not be a necessary structural element for regulating α-glycosidase and gluconeogenesis, which provided valuable information for the potential chemical modification of bioactive compounds (Atanasov et al., 2021; Attwood et al., 2021) (Figure 4B). Additionally, we noted that several natural products containing (non) polycyclic or unsaturated chain structures could regulate anti-oxidation, anti-inflammatory, glucose and lipid metabolism, and insulin function to achieve antidiabetic effects by enhancing the activity of antioxidant enzymes, downregulating inflammatory factors, and upregulating GLUT4 and PPAR-γ. On the one hand, these results indicated that such a polycyclic structure was an essential skeleton in treating diabetes. On the other hand, a few compounds with unsaturated chain structures (such as curcumin, cinnamaldehyde, and crocin) could also achieve antidiabetic effects *via* a similar mechanism (Figure 4C). This interesting phenomenon inspired us to hypothesize that fusing the polycyclic structure with an unsaturated chain moiety may benefit the discovery of novel antidiabetic drug candidates with synergistic therapeutic effects.

However, transforming natural products into clinical drugs still faces multiple challenges. For future research on discovering antidiabetic drugs, we propose the following directions: 1) The detailed mechanisms related to T2DM go far behind their chemical research. Therefore, in-depth pharmacodynamic studies are greatly needed to clarify the hypoglycemic mechanisms of these bioactive molecules. 2) Many experiments point out that the susceptibility of T2DM is highly associated with various genes (Langenberg and Lotta, 2018). The regulation of diabetes by natural active products at the genetic level may be a promising direction for research and the conduction of genetic experiments utilizing advanced technology, such as protein genomics research, may open a new avenue (Aslam et al., 2017). 3) The low water solubility significantly hinders the clinical application of several ingredients, such as flavonoids and terpenoids. One promising direction is to try to improve their bioavailability using chemical modification (Das et al., 2022) and nano-delivery systems (Al-Kassas et al., 2017). 4) Phytochemicals are a vital resource for discovering new drugs and sconducting the semi- or total synthesis and biotransformation of these antidiabetic components should be carried out in the future, which benefits the synthesis of a series of structural analogs and investigation of the structure-activity relationship.

Beyond these advancements, there are still many traditional medical systems worldwide that can effectively guide the application of bioactive metabolites in managing DM. For instance, Alexander N. Shikov et al. (2021) summarized the antidiabetic effects and characteristics of the primary metabolites in 227 Russian traditional medicinal plants. These compounds’ hypoglycemic mechanism and bioactivity prediction supported the rationality of multi-herbal mixtures in traditional Russian medicine. Similarly, under the guidance of TCM theory, the utilization of TCM and its metabolites in treating chronic diseases such as diabetes mellitus should also follow strict compatibility rules to achieve the superimposed therapeutic effect. In addition, due to the many natural products in medicinal plants and their complex derivatives, it is difficult to fully reveal their multiple hypoglycemic mechanisms through limited *in vivo* experiments or clinical trials. Therefore, it may be of great significance to further identify the extensive role of these metabolites in the treatment of DM by further strengthening the screening of bioactive metabolites and the synergistic mechanism of drug efficacy through advanced mathematical algorithms.
